# Closely-related taxa influence woody species discrimination via DNA barcoding: evidence from global forest dynamics plots

**DOI:** 10.1038/srep15127

**Published:** 2015-10-12

**Authors:** Nancai Pei, David L. Erickson, Bufeng Chen, Xuejun Ge, Xiangcheng Mi, Nathan G. Swenson, Jin-Long Zhang, Frank A. Jones, Chun-Lin Huang, Wanhui Ye, Zhanqing Hao, Chang-Fu Hsieh, Shawn Lum, Norman A. Bourg, John D. Parker, Jess K. Zimmerman, William J. McShea, Ida C. Lopez, I-Fang Sun, Stuart J. Davies, Keping Ma, W. John Kress

**Affiliations:** 1 State Key Laboratory of Tree Genetics and Breeding, Chinese Academy of Forestry, Beijing 100091, PR China; 2 Research Institute of Tropical Forestry, Chinese Academy of Forestry, Guangzhou 510520, PR China; 3 Department of Botany, MRC-166, National Museum of Natural History, Smithsonian Institution, P.O. Box 37012, Washington, DC 20013-7012, USA; 4 South China Botanical Garden, Chinese Academy of Sciences, Guangzhou 510650, PR China; 5 State Key Laboratory of Vegetation and Environmental Change, Institute of Botany, Chinese Academy of Sciences, Beijing 100093, PR China; 6 Department of Biology, University of Maryland, College Park, MD 20742, USA; 7 Flora Conservation Department, Kadoorie Farm and Botanic Garden, Lam Kam Road, Tai Po, N.T., Hong Kong; 8 Department of Botany and Plant Pathology, Oregon State University, 2082 Cordley Hall, Corvallis, OR, 97331, USA; 9 Laboratory of Molecular Phylogenetics, Department of Biology, National Museum of Natural Science, Taichung, Taiwan; 10 State Key Laboratory of Forest and Soil Ecology, Institute of Applied Ecology, Chinese Academy of Sciences, Shenyang 110016, PR China; 11 Institute of Ecology and Evolutionary Biology, National Taiwan University, Roosevelt Road 1, Taipei, Taiwan; 12 National Institute of Education of Nanyang Technological University, Singapore 637616; 13 Smithsonian Conservation Biology Institute, Front Royal, VA, USA; 14 Smithsonian Environmental Research Center, Edgewater, Maryland, USA; 15 Institute for Tropical Ecosystem Studies, University of Puerto Rico, San Juan Puerto Rico, 00936-8377, USA; 16 Department of Natural Resources and Environmental Studies, National Dong Hwa University, Hualien, Taiwan; 17 Center for Tropical Forest Science-Forest Global Earth Observatory, Smithsonian Tropical Research Institute, P.O. Box 37012, Washington, DC 20013-7012, USA

## Abstract

To determine how well DNA barcodes from the chloroplast region perform in forest dynamics plots (FDPs) from global CTFS-ForestGEO network, we analyzed DNA barcoding sequences of 1277 plant species from a wide phylogenetic range (3 FDPs in tropics, 5 in subtropics and 5 in temperate zone) and compared the rates of species discrimination (RSD). We quantified RSD by two DNA barcode combinations (*rbc*L + *mat*K and *rbc*L + *mat*K + *trn*H-*psb*A) using a monophyly-based method (GARLI). We defined two indexes of closely-related taxa (G_m_/G_t_ and S/G ratios) and correlated these ratios with RSD. The combination of *rbc*L + *mat*K averagely discriminated 88.65%, 83.84% and 72.51% at the local, regional and global scales, respectively. An additional locus *trn*H-*psb*A increased RSD by 2.87%, 1.49% and 3.58% correspondingly. RSD varied along a latitudinal gradient and were negatively correlated with ratios of closely-related taxa. Successes of species discrimination generally depend on scales in global FDPs. We suggested that the combination of *rbc*L + *mat*K + *trn*H-*psb*A is currently applicable for DNA barcoding-based phylogenetic studies on forest communities.

Species discrimination (or identification, delimitation) refers to recognizing biological units based on morphological, ecological or molecular characters, which is one of central topics to biology and particularly important for biodiversity conservation and evolutionary ecology^1^,^2^,^3^,^4^,^5^. The tropics and subtropics hold the majority of global biodiversity, containing 30 of the 34 biodiversity hotspots around the world (according to Conservation International up to year 2010)^6^,^7^. Theoretically and empirically, incorrect/ambiguous species discrimination could result in inaccurate knowledge about taxonomic classification^3^, biased estimations on ecological patterns and processes in biological communities^8^, and ineffective strategy-making for biodiversity conservation^5^, etc. Previous work has highlighted that most animal taxa can be identified to species level via DNA barcoding technology from a mitochondrial gene cytochrome *c* oxidase subunit 1 (*COI*)^9^,^10^, which indicates that DNA barcodes can solve the identification problem in biology-related fields. Furthermore, DNA barcoding approach works better but costs less than traditional morphology-based taxonomic practices for species discrimination in vegetation surveys^3^. Traditional plant taxonomy will encounter with practical difficulties when discriminating samples at early stage (e.g., shoots, seedlings, treelets) and/or only having an un-featured fragment (e.g., broken branches, dried stems, powdered leaves). More problems will arise, especially in the areas with masses of rare, not well-known, and potentially threatened plant species^11^. Therefore, detecting the general performance of plant DNA barcodes in discriminating species and exploring potential factors influencing discrimination rates are of significant importance for researches on biodiversity and ecology.

A two-locus combination of *rbc*L + *mat*K from the chloroplast region has been approved as a core DNA barcode to identify land plants, which achieves a rate of 72% correct species level identification based on a data set of 550 species representing the major lineages of land plants (including 445 angiosperm, 38 gymnosperm, and 67 cryptogam species)^12^. However, several empirical studies demonstrate that, the *trn*H-*psb*A intergenic spacer^13^,^14^, *ycf1* from the chloroplast region^15^, and nuclear ribosomal internal transcribed spacer (ITS) could also be complemented as the core DNA barcodes for seed plants^16^, in consideration of their inherent advantages (containing more evolutionary information) and disadvantages (different to amplify, unavailable to access sequence data for some forest plots at present). To date, most evaluations on DNA barcodes focused on particular taxonomic groups (taxon-level; family or genus), and usually came out a relatively weak discriminatory power^5^,^17^,^18^. In the family Combretaceae (63 species in six genera) in southern Africa, a two-locus core DNA barcode is found to perform poorly but the addition of *trn*H-*psb*A to work well, which indicate that evolutionary and biogeographic histories may influence the success of DNA barcoding in discriminating closely-related taxa^19^. However, for studies in the context of limited geographic forest regions (community-level) where many species from distantly related clades co-occur, it is likely that plant DNA barcoding can achieve a high rate of species discrimination^20^. Several studies have involved forest communities, but the rates of correct species identification by DNA barcodes are relatively inconsistent. In the two 1-ha plots of an Amazonian tropical forest in French Guiana, a TaxonDNA method achieved a rate of correct plant identification less than 70%, regardless of combinations of eight plant DNA markers tested in 252 juvenile tree species^21^. Meanwhile, with the aid of a supermatrix phylogeny-building approach, a BLAST method obtained much higher rates of correct species identification, via combinations of a two-locus core DNA barcode and a three-locus DNA barcode (*rbc*L + *mat*K + *trn*H-*psb*A) accordingly; greater than 92%/98% in the 50-ha BCI FDP in Panama^14^, 89%/93% in the 16-ha Luquillo FDP in Puerto Rico^22^, and 81%/87% in the 20-ha Dinghushan FDP in China^23^. Among a data set of 436 plant species (269 genera) in a temperate Canadian Koffler Scientific Reserve, rates of species discrimination were 100% for gymnosperms, bryophytes, lycophytes and monilophytes, compared with 92.7% in angiosperms using monophyly-based method^24^.

DNA barcoding is a powerful method in detecting new species even in well explored lineages and geographic areas under morphological scrutiny, with the aids of regional and international DNA barcoding libraries^25^. These remarkable findings inspire us to probe into the significance and effectiveness of plant DNA barcodes when discriminating species in forest communities. As more studies conducted using different methods, results of discrimination should reconcile with one another to move DNA barcoding method forward, and investigations on rates of correct species discrimination in diverse forest communities could be applied across multiple ecosystems and forest types. Furthermore, we are eager to detect possibly general patterns of plant DNA barcodes performing on more diverse taxonomic groups and at larger geographic scales worldwide, and try to explore potential factors influencing discrimination rates. Our work does not solely focus on issues of taxonomy, but dedicates to provide a new pathway to discriminate plant taxa sampled in forest communities using the universal DNA barcoding method, which will hopefully facilitate species classification and be applied to related research fields, especially in an era of shortage of taxonomists specialized on large-scale floristic studies.

Forest dynamic plots (FDPs) provide a unique opportunity to study large-scale research and multidisciplinary researches on forests including DNA barcoding. DNA barcoding libraries based on FDPs could then provide a non-distorted picture. To the best of our knowledge, the present study comprehensively reported a general performance of the suggested two-locus core DNA barcode of land plants on tree species discrimination, and the improvements when adding a complementary DNA barcode (i.e., *trn*H-*psb*A spacer region) using a monophyly-based method (i.e., GARLI), utilizing large data sets from 13 CTFS-ForestGEO FDPs ranging from tropics to temperate zones. The present study included three objects. Firstly, we compared rates of species discrimination using two DNA barcode combinations and along a latitudinal gradient. GARLI method was utilized to calculate rates of species discrimination when using DNA barcoding sequence data. Secondly, we have regressed closely-related taxa ratios (S/G and G_m_/G_t_ ratios) against a latitudinal gradient. Two indexes of closely-related taxa were used; specifically, ratio of multiple-species genus/total genera (G_m_/G_t_ ratio: the number of multiple-species genus (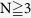 species per genus) to the total number of genera), and species/genus ratio (S/G ratio: the number of species per genus). These ratios were mapped accordingly along a latitudinal gradient. Finally, we correlated ratios of closely related taxa with rates of species discrimination. We predicted that the accuracy of correct species discriminations using plant DNA barcodes was mainly determined by the ratios of closely-related taxa (i.e., S/G ratio and G_m_/G_t_ ratio) in global FDPs.

## Results

### Rates of species discrimination

**Figure 1:**
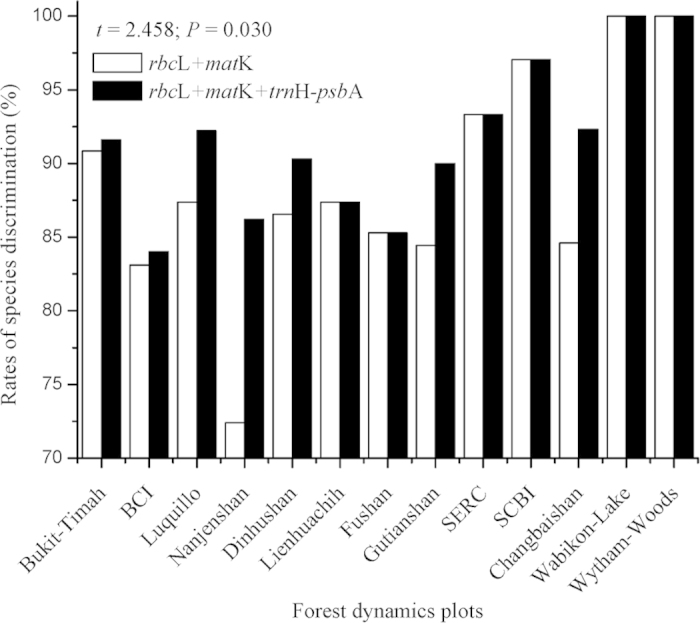
Species discrimination rates of *rbc*L + *mat*K and *rbc*L +  *mat*K + *trn*H–*psb*A among the thirteen forest dynamics plots.

Locally (n = 13), average rates of species discrimination (RSDs) were 88.64 ± 2.12% for *rbc*L + *mat*K and 91.52 ± 1.44% for *rbc*L + *mat*K + *trn*H-*psb*A; mean ± S.E.) (Fig. 1). For *rbc*L + *mat*K, RSD was the highest in Wabikon-Lake (100%) and Wytham-Woods (100%) from the temperate zone, but the lowest in Nanjenshan (72.41%) from the subtropics. For *rbc*L + *mat*K + *trn*H-*psb*A, RSD was also the highest in Wabikon-Lake (100%) and Wytham-Woods (100%), but the lowest in BCI (84%) from the tropics. Regionally (n = 3), average RSDs were 83.84 ± 8.11% for *rbc*L + *mat*K and 83.33 ± 7.35% for *rbc*L + *mat*K + *trn*H-*psb*A. RSD was the highest in the temperate zone (100%) both for *rbc*L + *mat*K and *rbc*L + *mat*K + *trn*H-*psb*A, but the lowest in the subtropics (74.46% for *rbc*L + *mat*K and 77.23% for *rbc*L + *mat*K + *trn*H-*psb*A). Globally (among all the species) (n = 1), RSDs were 75.51% for *rbc*L + *mat*K and 76.09% for *rbc*L + *mat*K + *trn*H-*psb*A.

### Improvements of species discrimination by adding *trn*H-*psb*A to *rbc*L + *mat*K combination

An additional DNA barcoding locus (*trn*H-*psb*A) had increased rates of species discrimination to various degrees. Specifically, average RSDs increased from 88.65 ± 7.65% to 91.52 ± 5.19% (*t* = 2.458, *P* = 0.030, n = 13) locally, from 83.84 ± 8.11% to 85.33 ± 7.35% (*t* = 1.848, *P* = 0.206, n = 3) regionally, and from 72.51% to 76.09% globally (n = 1). Furthermore, the discrimination of all the plots united without multiple-species genera increased to 84.46% when compared to that with multiple-species genera.

### Species discrimination along a latitudinal gradient

**Figure 2:**
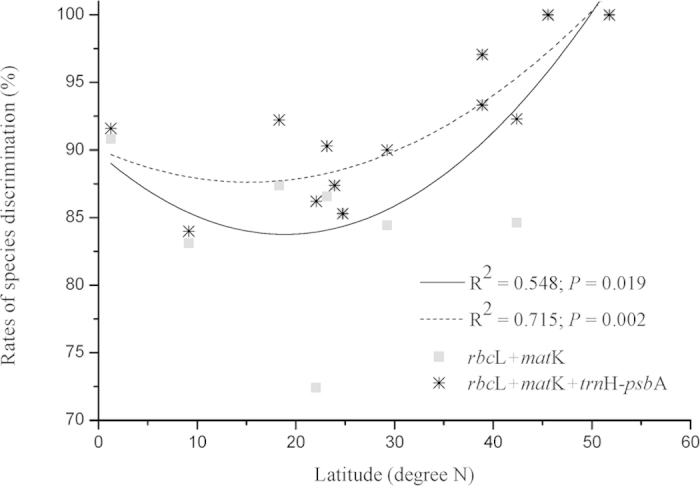
Species discrimination rates of *rbc*L + *mat*K and *rbc*L + *mat*K + *trn*H–*psb*A along a latitudinal gradient.

Determination coefficients (R^2^) values were 0.548 (*P* = 0.019) and 0.715 (*P* = 0.002) for *rbc*L + *mat*K and *rbc*L + *mat*K + *trn*H-*psb*A with latitudinal factors under a quadratic model (Fig. 2), while only 0.294 and 0.504 when under a linear model. Correlation coefficients (Pearson’s *r*) were 0.740 and 0.846 under quadratic models, indicating a relatively high correlation. Rates of species discrimination varied along a latitudinal gradient, which tended to decrease from low to middle latitudes, and to increase from middle to high latitudes. Consequently, a quadratic regression model seems to be more appropriate when describing distribution patterns of species discrimination rates along a latitudinal gradient.

### A latitudinal gradient of closely-related taxa ratios (S/G and G_m_/G_t_ ratios)

S/G ratios ranged from 1.000 to 1.750 (on average 1.466) among all the 13 plots; specifically from 1.316 to 1.614 (on average 1.466) in tropics, from 1.304 to 1.714 (on average 1.554) in subtropics, and from 1.000 to 1.750 (on average 1.378) in temperate zones. S/G ratios in subtropics were higher than in tropics and temperate zones, but no statistical significance could be detected (*F* = 0.835, *P* = 0.462). G_m_/G_t_ ratios ranged from 0 to 0.178 (on average 0.095) among all the 13 plots; specifically from 0.076 to 0.100 (on average 0.092) in tropics, from 0.043 to 0.178 (on average 0.123) in subtropics, and from 0 to 0.150 (on average 0.069) in temperate zones. G_m_/G_t_ ratios in subtropics were higher than in tropics and temperate zones, but no statistical significance could be detected (*F* = 1.309, *P* = 0.313). Determination coefficients (R^2^) were 0.362 and 0.390 for S/G and G_m_/G_t_ ratios with latitudinal factors under a quadratic regression model, while only 0.142 and 0.134 when under a linear model. Consequently, both S/G ratios and G_m_/G_t_ ratios tended to increase from low to middle latitudes, and to decrease from middle to high latitudes. Correlation coefficients (*r*) were 0.602 and 0.624 for S/G and G_m_/G_t_ ratios with latitudinal factors using a quadratic model, indicating a moderate correlation.

### Correlations of closely-related taxa ratios with rates of species discrimination

**Figure 3:**
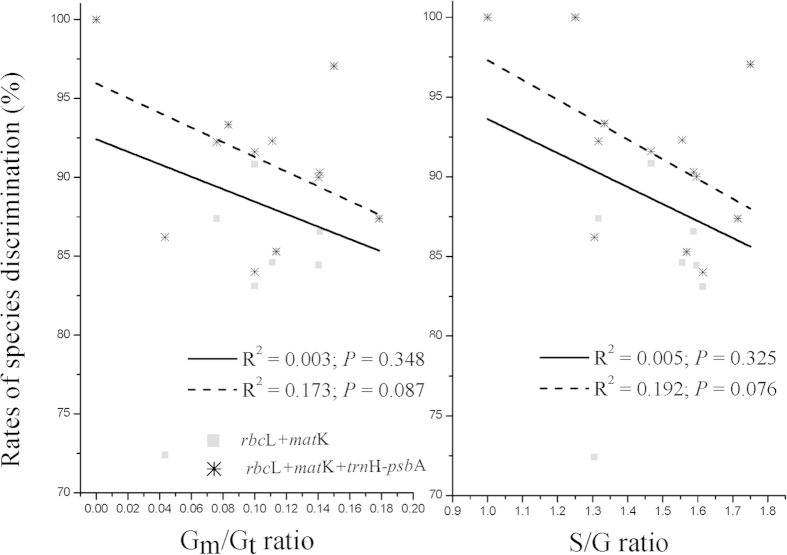
Correlations of closely-related taxa ratios with species discrimination rates of *rbc*L + *mat*K and *rbc*L +  *mat*K + *trn*H–*psb*A (left side: G_m_/G_t_ ratios; right side: S/G ratios).

RSDs tended to be higher in FDPs with lower ratios of closely-related taxa (S/G and G_m_/G_t_ ratios), and to be lower in FDPs with higher ratios. R^2^ values varied from 0.005 to 0.192 for S/G ratio and from 0.003 to 0.173 for G_m_/G_t_ ratio when using a linear regression model (Fig. 3). Rates of species discrimination were negatively correlated with ratios of closely-related taxa, but no statistical significance was detected (all *P* > 0.05).

## Discussion

A central goal of this work is to assess how well DNA barcodes perform in global FDPs by comparing the success of species correct discrimination. Higher rates of species discrimination in forest communities can improve the resolution of community phylogenies, thus phylogenetically related biodiversity indexes could be more accurate, and the mechanisms governing the community assembly across large spatio-temporal scales could be more clearly inferred^14^,^26^,^27^,^28^,^29^. Many fields of the natural sciences are declining, especially traditional disciplines like taxonomy^30^. Taxonomists have become an endangered profession in the era of genomics over the past few decades, but now taxonomy suddenly becomes fashionable again due to revolutionary methods in taxonomy called DNA barcoding. Integrative taxonomy incorporates various types of data and quantitative methods for documenting biodiversity and facilitates the integration of data from different sources^31^,^32^. With the development of universal loci, standard working flow, complete taxonomic sampling, and rapid species discrimination, DNA barcoding may invigorate traditional disciplines in the context of local, regional and global scales^29^,^33^,^34^. Certainly, genetic data like DNA barcoding sequences should be used with caution, since there is no common distance threshold identified for species delimitation, no single classification technique applied universally for species identification, and practical difficulties to discriminate closely-related species adopting genetic distance methods. Hence, we need corroboration from ecology, morphology, geography, and molecular information, especially the case in the context of species discovery^35^,^36^. The data sets used in the study (13 FDPs from the CTFS-ForestGEO network) at least provide a good opportunity to describe general patterns of tree species discriminations using plant DNA barcodes at the local, regional and global scales, and to detect how potential factors (e.g., S/G ratio, G_m_/G_t_ ratio, and latitudes) influence rates of species discrimination.

For species discrimination in diverse forest communities, plant DNA barcoding satisfies criterions of simplicity, repeatability, effectiveness, and standardization. GARLI method had a relatively high rates of species discrimination (88.64–91.52%) among the 13 FDPs (Fig. 1), suggesting that the monophyly-based method distinguished excellent in terms of species discrimination, though monophyly-based methods using DNA barcodes has been criticized because it assumes that phylogenetic reconstruction is reliable^21^. Our results showed that *rbc*L, *mat*K, and *trn*H-*psb*A could be treated as most proper (though not ideal) genetic regions for plant DNA barcoding studies in forest communities. Besides of monophyly-based method, distance and character-based methods is also proposed to classify species through a unique combination of diagnostic character states from molecular sequences that have been organized hierarchically^36^,^37^,^38^, which may perform well for species identification of populations and genera. None method was found to be the best to discriminate plant species, and a feasible option could be “the simplest, the best” in diverse FDPs.

Numerous studies have found that *rbc*L + *mat*K results in low rates of species discrimination in taxonomic groups (i.e., at the taxon-level), because this combination is limited in its ability to correctly identify species due to low levels of sequence variability in these two chloroplast gene regions. CBOL Plant Working Group has reported a general rate of ~72% successful discrimination rate based on a comprehensive analysis across 550 species representing the major lineages of land plants^12^. Sometimes, these low rates of species discrimination can be extremely variable; for example, it is only 26% of RSD in *Rhododendron* with 173 species^39^, 73% RSD in *Taxus* covering all species in Eurasia^40^, depending on the particular taxa examined. However, this two-locus core DNA barcode performs much better in geographically restricted regions (i.e., at the community level), especially in forest dynamics plots. In temperate zone, a national DNA barcode resource covering the native flowering plants and conifers for Wales, provides discrimination rates of 1143 species ranging from 69.4% to 74.9%^41^. In a Canadian Arctic flora, a distance-based analysis of combined *rbc*L + *mat*K sequence data discriminates 56% of 490 vascular plant species^42^, while at the Koffler Scientific Reserve, Canada, the *rbc*L + *mat*K DNA barcode can result in 93.1% of species resolution in the context of local northern temperate floras using monophyly-based method^24^, which could be caused by the different calculation methods. In an African rainforest plot in Cameroon, a BLAST analysis discriminates 80% of 272 species with the *rbc*L + *mat*K combination^43^. It’s reasonable that *rbc*L + *mat*K can obtain relatively high rates of species discrimination in diverse FDPs at local scale, which can be largely explained by the presence of a broad taxonomic sampling and relatively few closely related taxa co-occurring in the geographically restricted region^20^,^44^.

The strategy combining one locus with slower evolutionary rate plus another one or two loci with faster evolutionary rate, utilizing markers from chloroplast (e.g., *rbc*La/*rbc*Lb, *mat*K, and *trn*H-*psb*A) and/or nuclear genes (e.g., ITS/ITS2), could be used as an effective DNA barcode to discriminate species for land plants (at the taxon or community levels), depending on the phylogenetic information from the study sites and ratios of closely-related taxa. The addition of a non-coding region *trn*H-*psb*A to the core DNA barcode increased species resolution by 2.2% (to 95.3% at the Koffler Scientific Reserve, Canada)^24^, by 4% (to 93% in the 16-ha Luquillo FDP in Puerto Rico)^22^, by 6% (to 87% in the 20-ha Dinghushan FDP in China and to 98% in the 50-ha BCI FDP in Panama)^14^,^23^, and by 8% (to 88% in a 50-ha FDP in Cameroon)^43^. However, our results found that a complementary DNA barcode (i.e., *trn*H-*psb*A) could averagely improve RSDs by 2.9% (to 91.5% using GARLI method) for the 13 FDPs (Fig. 1), especially high in subtropical regions. A meta-analysis of DNA sequences from 3495 species of land plants belonging to 498 genera in 149 families searched from GenBank finds that single *trn*H-*psb*A results in a relative low rate of species discrimination (from 35.5% in gymnosperms to 72.2% in mosses) using BLAST method, while *trn*H-*psb*A + ITS2 combination performs better or equally well compared with other combinations (e.g., *trn*H-*psb*A, *mat*K, *rbc*L, and ITS2 from 586 species belonging to 71 genera and 47 families) in most taxonomic groups studied^45^. The addition of ITS/ITS2 is more powerful in specific taxonomic groups due to its faster evolutionary rate than *trn*H-*psb*A^44^,^46^.

Species-genus (S/G) ratio and multiple-species genus to the total genera (G_m_/G_t_) ratio are sensitive to diversity and may indicate recent speciation in a given area^47^,^48^, which is hoped to serve as a potential proxy indicator to describe the phylogenetic relatedness of targeted clades in a biological community. S/G ratios are expected to present a strong latitudinal gradient correlation at the global scale, with high ratios in tropics and low ratios in temperate zones^49^. Similarly, a significant negative correlation was found between the number of species per genus and the rate of species discrimination per genus in a Canadian Arctic flora^42^, and a weak relation in a Canadian temperate flora^24^. Our results partially supported the prediction of latitudinal diversity gradient, but with an exception in subtropical region, which may relate to speciation and range-expansion events. Furthermore, our results found that rates of species discriminations negatively correlated with ratios of closely related taxa (S/G and G_m_/G_t_ ratios) across global CTFS-ForestGEO FDPs (Fig. 3).

Species diversity and congener number decrease with the rising of the latitudinal gradient^50^,^51^. Our results showed that a nonlinear trend of species discrimination rates varied along a latitudinal gradient, which tended to decrease from low to middle latitudes, and to increase from middle to high latitudes, indicating a turning point in subtropics (Fig. 2). Consequently, a quadratic regression model rather than linear or other types of regression models (with higher coefficients of determination), was probably proper to describe distribution patterns of species discrimination rates along a latitudinal gradient. The turning point in subtropical region was likely attributed to insufficient sampling, though a comprehensively up-to-date dataset of 13 FDPs had been included in the study. Likewise, the turning point could also be explained by particular species composition and geographical characteristics. Subtropical China (including mainland and Taiwan Island) cover diverse vegetation types and endemic plant species^52^,^53^,^54^, which may undergo regional complex climate changes and local environmental factors throughout the last ice-age cycles from individual to ecosystem. The relatively low rates of species discrimination in subtropical FDPs could be caused by cryptic plant diversity and endemism in these refuge areas^55^. Species assembly in local communities (e.g., species number in a specific genus, or the number of multiple-species genus) is relevant to deterministic (e.g., species traits, interspecies interactions, and environmental conditions) and/or stochastic processes (e.g., birth, death, colonization, extinction, and speciation)^56^,^57^,^58^.

ITS/ITS2 from nuclear gene region will definitely provide more phylogenetic information for community studies, and is also expected to play an important role to discriminate closely-related taxa in taxonomic groups. The combination of *trn*H-*psb*A + ITS and *rbc*L + *mat*K + ITS (ITS2) was suggested as the preferred barcodes for tropical and subtropical tree species identification in natural reserve, respectively^44^,^59^. In conclusion, based on the comprehensive consideration among the rates of species discrimination, cost-effectiveness, sequence recovery and alignment, and phylogenetic reconstruction, we suggested that a three-locus DNA barcode (*rbc*L + *mat*K + *trn*H-*psb*A) from chloroplast gene region is effective and proper for studies on plant communities, especially in CTFS-ForestGEO FDPs (Table 1).

## Methods

### Selected forest dynamics plots

Thirteen FDPs ranging from 2 to 50-ha of CTFS-ForestGEO network (http://www.ctfs.si.edu/) across diverse forests were included in the present study (Supplementary Table S1). These plots span c. 50 degrees of latitude across the globe. All the FPDs were set up according to the standard Smithsonian CTFS protocol^60^. This work was conducted based on Forestry Standards for “Observation Methodology for Long-term Forest Ecosystem Research” of the People’s Republic of China (LY/T 1952–2011).

### DNA barcode libraries

Three standard DNA barcodes from the chloroplast region (i.e., *rbc*L, *mat*K and *trn*H-*psb*A) were used to obtain the sequence data for plant species in each plot^61^ (Supplementary Table S2). All the 13 FDPs contained 1227 plant species (excluding repeated species), comprising samples with a high-quality sequence for at least one of the three regions. To facilitate comparative analyses, we obtained 17 subsets with sequences for all the three gene regions: 13 individual FDPs with 8 to 226 species at the local scale; tropical, subtropical and temperate regions with 472, 326, and 106 species at the regional scale; and all the FDPs united with 896 species (excluding repeated species) accompanied with DNA barcode sequences from three loci simultaneously at the global scale.

The selected 896 plant species accompanied with DNA sequences from the three loci simultaneously, encompassing 428 genera in 105 families and 37 orders and varying from 8 to 226 species in multiple FDPs. For each forest plot, ten variables were collected, namely latitude, climatic region, hemisphere, plot size, number of genus, number of species, number of multiple-species genus, S/G ratio, G_m_/G_t_ ratio, and rates of species discrimination.

### Rates of species discrimination

Rates of species discrimination (RSD) for all data sets were obtained using monophyly-based method. The phylogenetic relationship was inferred by maximum likelihood method using GARLI (Genetic Algorithm for Rapid Likelihood Inference) available on CIPRES (http://www.phylo.org/portal2/updateProfile.action) for each barcoding library. The above method allows sequences of three gene regions combined as a matrix, which were not feasible in BLAST method. All DNA sequence data were kept as FASTA format, and were aligned as NEXUS format using NCL Converter on CIPRES for subsequent phylogenetic tree reconstruction. The numbers of monophyletic lineages showed how well a phylogenetic tree was reconstructed. More details about GARLI method and analyses can be found in a previous literature^61^.

### Data analyses

To test how much can be improved when adding the third locus *trn*H-*psb*A to the core DNA barcode (*rbc*L + *mat*K) for land plants, two DNA barcode combinations were used for all the analyses at the local, regional and global scales. Oneway ANOVA and Tukey Post Hoc Tests were used to illustrate differences between the discrimination rates. To detect the trend of species discrimination rates along a latitudinal gradient, several regression models were compared. The one with the highest determination coefficient (R^2^) was selected as the best fit model.

## Additional Information

**How to cite this article**: Pei, N. *et al*. Closely-related taxa influence woody species discrimination via DNA barcoding: evidence from global forest dynamics plots. *Sci. Rep*. **5**, 15127; doi: 10.1038/srep15127 (2015).

## Supplementary Material

Supplementary Information

## Figures and Tables

**Table 1:** Comparisons of performance and evaluation (cost-effectiveness) of commonly used combinations of plant DNA barcodes, according to published literatures cited in this study.

Items	*rbc*L + *mat*K	*rbc*L + ITS/ITS2	*rbc*L + *mat*K + *trn*H-*psb*A	*rbc*L + *mat*K + ITS/ITS2	*rbc*L + *mat*K + *trn*H-*psb*A + ITS/ITS2
Sequence recovery and alignment	Easy	Difficult	Medium	Difficult	Difficult
Rates of species discrimination	Low (taxon-level); Moderate (community-level)	Low (taxon-level); Moderate (community-level)	Low (taxon-level); High (community-level)	Low (taxon-level); High (community-level)	− (Estimated to be high)
Community phylogenetic reconstruction	Poorly-resolved	–	Well-resolved	–	− (Estimated to be well-resolved)
Cost	Less	Less	Medium	Medium	More

Rates of species discrimination: “Low” (<75%); “Moderate” (75–90%); “High” (>90%).

Community phylogenetic reconstruction: “Poorly-resolved” (<75%); “Well-resolved” (>90%).

“–”: not reported.
