# Supplementary material for: Closely-related taxa influence woody species discrimination via DNA barcoding: evidence from global forest dynamics plots
Source: Sci Rep. 2015 Oct 12;5:15127. doi: 10.1038/srep15127 (PMC4601009; doi:10.1038/srep15127)
Supplement: Supplementary Information [file srep15127-s1.pdf]

Closely-related taxa influence woody species discrimination via DNA barcoding: evidence from global forest dynamics plots

Nancai Pei[1,2*], David L. Erickson[3], Bufeng Chen[1,2], Xuejun Ge[4], Xiangcheng Mi[5], Nathan G. Swenson[6], Jin-Long Zhang[7], Frank A. Jones[8], Chun-Lin Huang[9], Wanhui Ye[4], Zhanqing Hao[10], Chang-Fu Hsieh[11], Shawn Lum[12], Norman A. Bourg[13], John D. Parker[14], Jess K. Zimmerman[15], William J. McShea[13], Ida C. Lopez[3], I-Fang Sun[16], Stuart J. Davies[17], Keping Ma[5] & W. John Kress[3*]

Supplementary Table S1

Description of the thirteen forest dynamics plots examined in this study. Totals are for the number of taxa (species and genera) among tropical, subtropical and temperate regions, and all plots united; and the non-redundant totals count each species only once.

Local/regional/global scales	Coordinates (Latitude/Longitude)	Climatic region	Hemisphere	Plot size (ha)	Number of species	Number of genus	Number of multiple-species genus (≧3 species/genus)	Ratio of multiple-species genus /total genera	Species/genus ratio
Bukit-Tima	1.25/103.75	Tropics	Asia	2	132	90	9	0.1	1.466666667
BCI	9.1543/-79.8461	Tropics	Latin America	50	226	140	14	0.1	1.614285714
Luquillo	18.3262/-65.816	Tropics	Latin America	16	104	79	6	0.075949367	1.316455696
Nanjenshan	22.059/120.854	Subtropics	Asia	3	30	23	1	0.043478261	1.304347826
Dinhushan	23.1558/112.511	Subtropics	Asia	20	135	85	12	0.141176471	1.588235294
Lienhuachih	23.9136/120.879	Subtropics	Asia	25	96	56	10	0.178571429	1.714285714
Fushan	24.7614/121.555	Subtropics	Asia	25	69	44	5	0.113636364	1.568181818
Gutianshan	29.2503/118.119	Subtropics	Asia	24	91	57	8	0.140350877	1.596491228
SERC	38.889/-76.559	Temperate zone	North America	16	16	12	1	0.083333333	1.333333333
SCBI	38.8916/-78.1457	Temperate zone	North America	25.6	35	20	3	0.15	1.75

Changbaishan	42.3833/128.083	Temperate zone	Asia	25	14	9	1	0.111111111	1.555555556
Wabikon-Lake	45.5508/-88.7964	Temperate zone	North America	25.6	10	8	0	0	1.25
Wytham-Woods	51.7743/-1.3379	Temperate zone	Europe	18	8	8	0	0	1
Tropics					472	183	38	0.207650273	2.579234973
Subtropics					326	153	34	0.222222222	2.130718954
Temperate zone					106	52	12	0.230769231	2.038461538
All the plots united					896	428	83	0.193925234	2.093457944

Supplemental Table S2

Names and Accession numbers for all plant species in the study. Listed by presence in CTFS-ForestGEO forest dynamics plots and alphabetically by taxonomy. Items without GenBank accession numbers could be retrievable when queried.

Taxon	Family	Site	*rbc*L	*mat*K	*trn*H-*psb*A
Abarema macradenia	Fabaceae	BCI	GQ981652	GQ981925	GQ982133
Abies balsamea	Pinaceae	SERC	KJ593167	n/a	n/a
Abies holophylla	Pinaceae	Changbaishan	CANGB001-14	CANGB001-15	CANGB001-16
Abies nephrolepis	Pinaceae	Changbaishan	CANGB002-14	CANGB002-15	CANGB002-16
Acalypha diversifolia	Euphorbiaceae	BCI	GQ981653	n/a	GQ982134
Acalypha macrostachya	Euphorbiaceae	BCI	GQ981654	GQ981926	GQ982135
Acer barbinerve	Sapindaceae	Changbaishan	CANGB003-14	CANGB003-15	CANGB003-16
Acer campestre	Sapindaceae	Wytham	KM360609	FJ395381	FJ395476
Acer ginnala	Sapindaceae	Changbaishan	CANGB004-14	n/a	n/a
Acer mandshuricum	Sapindaceae	Changbaishan	CANGB005-14	CANGB005-15	CANGB005-16
Acer mono	Sapindaceae	Changbaishan	CANGB006-14	CANGB006-15	CANGB006-16
Acer negundo	Sapindaceae	SERC	HM849738	EU749281	DQ006227
Acer olivaceum	Sapindaceae	Gutianshan	HQ427190	HQ427338	HQ427032
Acer pseudoplatanus	Sapindaceae	Wytham	HE963306	n/a	HE966461
Acer pseudosieboldianum	Sapindaceae	Changbaishan	CANGB007-14	CANGB007-15	CANGB007-16
Acer rubrum	Sapindaceae	SERC	KJ593173	KJ592831	HQ266324
Acer saccharum	Sapindaceae	SERC	KJ593174	EU749290	EU750438
Acer tegmentosum	Sapindaceae	Changbaishan	CANGB008-14	CANGB008-15	CANGB008-16
Acer triflorum	Sapindaceae	Changbaishan	CANGB009-14	CANGB009-15	CANGB009-16
Acer ukurunduense	Sapindaceae	Changbaishan	CANGB010-14	n/a	n/a
Acer wilsonii	Sapindaceae	Gutianshan	HQ427189	HQ427337	HQ427031

Acronychia pedunculata	Rutaceae	Dinghushan	HQ415180	HQ415351	HQ415529
Actinodaphne malaccensis	Lauraceae	Bukit-Timah	KJ594563	KJ708793	BTSIN145-13
Adelia triloba	Euphorbiaceae	BCI	GQ981655	GQ981927	GQ982136
Adenanthera malayana	Fabaceae	Bukit-Timah	KJ594564	KJ708795	BTSIN315-13
Adenanthera microsperma	Fabaceae:Mimos.	Dinghushan	HQ415090	HQ415273	HQ415441
Adinandra dumosa	Pentaphylacaceae	Bukit-Timah	Z83149	KJ708799	BTSIN588-13
Adinandra integerrima	Pentaphylacaceae	Bukit-Timah	n/a	KJ708801	BTSIN599-13
Adinandra milletii	Pentaphylacaceae	Gutianshan	HQ427223	HQ427369	n/a
Aeschynomene americana	Fabaceae	Luquillo	AF30870	n/a	n/a
Aglaia elliptica	Meliaceae	Bukit-Timah	AY128210	KJ708802	BTSIN223-13
Aglaia exstipulata	Meliaceae	Bukit-Timah	KJ594567	KJ708803	BTSIN451-13
Aglaia glabriflora	Meliaceae	Bukit-Timah	KJ594568	KJ708804	n/a
Aglaia rufinervis	Meliaceae	Bukit-Timah	KJ594573	KJ708809	BTSIN292-13
Aglaia sexipetala	Meliaceae	Bukit-Timah	n/a	KJ708810	BTSIN647-13
Aidia canthioides	Rubiaceae	Dinghushan	HQ415144	n/a	HQ415494
Aidia cochinchinensis	Rubiaceae	Gutianshan	HQ427198	HQ427347	HQ427041
Aidia wallichiana	Rubiaceae	Bukit-Timah	KJ594574	KJ708811	BTSIN392-13
Alangium longiflorum	Cornaceae	Bukit-Timah	KJ594578	KJ708815	BTSIN196-13
Alangium nobile	Cornaceae	Bukit-Timah	KJ594579	KJ708816	BTSIN296-13
Albizia julibrissin	Fabaceae	SERC	GU135262	EU812010	EU811952
Albizia kalkora	Fabaceae:Mimos.	Gutianshan	HQ427141	HQ427295	HQ426986
Albizia splendens	Fabaceae	Bukit-Timah	BT-R01	KJ708817	BTSIN198-13
Alchornea costaricensis	Euphorbiaceae	BCI	JQ591406	n/a	n/a
Alchornea latifolia	Euphorbiaceae	BCI/Luquillo	HM446755	HM641813	HM446884
Alchornea trewioides	Euphorbiaceae	Dinghushan	HQ415177	HQ415348	HQ415526
Alchorneopsis floribunda	Euphorbiaceae	Luquillo	HM446756	HM446655	HM446885
Aleurites montana	Euphorbiaceae	Lienhuachih	KJ687971	KJ687450	KJ686651
Alfaropsis roxburghiana	Juglandaceae	Dinghushan	HQ415111	HQ415289	HQ415461
Alibertia edulis	Rubiaceae	BCI	GQ981657	GQ981930	GQ982139
Allophylus	Sapindaceae	BCI	GQ981658	JQ589126	GQ982140

psilospermus					
Alniphyllum fortunei	Styracaceae	Gutianshan	HQ427122	HQ427279	HQ426969
Alniphyllum pterospermum	Styracaceae	Fushan	KJ687974	n/a	n/a
Alnus incana	Betulaceae	Wabikon Lake	KJ593202	KJ592842	FJ011870
Alnus mandshurica	Betulaceae	Changbaishan	CANGB011-14	CANGB011-15	CANGB011-16
Alphonsea johorensis	Annonaceae	Bukit-Timah	BT-R02	KJ708818	BTSIN323-13
Alseis blackiana	Rubiaceae	BCI	GQ981659	n/a	GQ982141
Amaioua corymbosa	Rubiaceae	BCI	GQ981660	GQ981931	GQ982142
Amelanchier arborea	Rosaceae	SERC	JQ391252	JQ390935	JQ390642
Amelanchier laevis	Rosaceae	Wabikon Lake	HQ589949	n/a	n/a
Anacardium excelsum	Anacardiaceae	BCI	GQ981661	GQ981932	GQ982143
Anaxagorea panamensis	Annonaceae	BCI	GQ981662	GQ981933	GQ982144
Andira inermis	Fabaceae:Papil.	BCI/Luquillo	HM446757	GQ429072	HM446886
Anisoptera laevis	Dipterocarpaceae	Bukit-Timah	KJ594582	AB006370	BTSIN070-13
Anisoptera megistocarpa	Dipterocarpaceae	Bukit-Timah	KJ594584	KJ708822	BTSIN034-13
Annona acuminata	Annonaceae	BCI	GQ981664	GQ981934	GQ982146
Annona purpurea	Annonaceae	BCI	JQ590160	JQ586490	n/a
Annona spraguei	Annonaceae	BCI	GQ981665	GQ981935	GQ982147
Antiaris toxicaria	Moraceae	Bukit-Timah	KJ594588	GQ434236	BTSIN053-13
Antidesma bunius	Phyllanthaceae	Dinghushan	HQ415206	n/a	HQ415553
Antidesma coriaceum	Phyllanthaceae	Bukit-Timah	KJ594590	n/a	BTSIN089-13
Antidesma japonicum var. acutisepalum	Phyllanthaceae	Fushan/Lienhuachih	AB925514	n/a	KJ686654
Antidesma japonicum var. densiflorum	Phyllanthaceae	Fushan	KJ687977	n/a	KJ686655
Antidesma neurocarpum	Phyllanthaceae	Bukit-Timah	KJ594591	KJ708824	n/a
Antidesma venosum	Phyllanthaceae	Dinghushan	HQ415207	HQ415372	HQ415554
Antirhea obtusifolia	Rubiaceae	Luquillo	HM446758	HM446656	HM446887

Apeiba membranacea	Malvaceae	BCI	GQ981666	n/a	n/a
Apeiba tibourbou	Malvaceae	BCI	GQ981667	GQ981936	GQ982148
Aphelandra sinclairiana	Acanthaceae	BCI	GQ981668	GQ981937	GQ982149
Aporusa benthamiana	Phyllanthaceae	Bukit-Timah	KJ594594	KJ708826	BTSIN200-13
Aporusa falcifera	Phyllanthaceae	Bukit-Timah	KJ594596	n/a	n/a
Aporusa frutescens	Phyllanthaceae	Bukit-Timah	KJ594599	KJ708827	BTSIN457-13
Aporusa lunatum	Phyllanthaceae	Bukit-Timah	KJ594600	KJ708829	n/a
Aporusa miqueliana	Phyllanthaceae	Bukit-Timah	KJ594603	KJ708831	BTSIN469-13
Aporusa nervosa	Phyllanthaceae	Bukit-Timah	KJ594605	n/a	BTSIN235-13
Aquilaria sinensis	Thymelaeaceae	Dinghushan	HQ415056	HQ415244	HQ415408
Aralia chinensis	Araliaceae	Gutianshan	HQ427250	HQ427393	HQ427093
Aralia decaisneana	Araliaceae	Fushan	KJ687982	KJ687454	KJ686659
Aralia spinifolia	Araliaceae	Dinghushan	HQ415059	HQ415247	HQ415411
Archidendron clypearia	Fabaceae	Bukit-Timah	n/a	KJ708833	n/a
Archidendron lucidum	Fabaceae-Mimosoideae	Dinghushan/ Fushan	HQ415101	HQ415282	HQ415452
Archidendron turgidum	Fabaceae	Dinghushan	HQ415094	n/a	HQ415445
Ardisia bartlettii	Primulaceae	BCI	GQ981669	GQ981938	GQ982150
Ardisia colorata	Primulaceae	Bukit-Timah	KJ594606	KJ708834	BTSIN262-13
Ardisia cornudentata subsp. morrisonensis	Primulaceae	Fushan	KJ687988	n/a	n/a
Ardisia crenata	Primulaceae	Gutianshan/ Fushan	HQ427270	HQ427412	HQ427113
Ardisia glauca	Primulaceae	Luquillo	KJ082123	KJ012464	HM446888
Ardisia guianensis	Primulaceae	BCI	GQ981670	GQ981939	GQ982151
Ardisia quinquegona	Primulaceae	Dinghushan/ Fushan	HQ415236	HQ415400	HQ415582
Ardisia sieboldii	Primulaceae	Fushan	KJ688696	KJ687459	KJ686669
Ardisia standleyana	Primulaceae	BCI	GQ981671	GQ981940	GQ982152
Ardisia teysmanniana	Primulaceae	Bukit-Timah	KJ594608	n/a	BTSIN368-13
Ardisia virens	Primulaceae	Fushan	KJ687998	KJ687460	KJ686673
Arthrophyllum diversifolium	Araliaceae	Bukit-Timah	KJ594609	KJ708836	n/a
Artocarpus altilis	Moraceae	Luquillo	HM446760	HM446658	HM446889
Artocarpus anisophyllus	Moraceae	Bukit-Timah	KJ594612	n/a	BTSIN155-13

Artocarpus dadah	Moraceae	Bukit-Timah	KJ594613	n/a	BTSIN170-13
Artocarpus elasticus	Moraceae	Bukit-Timah	KJ594614	KJ708837	BTSIN424-13
Artocarpus hispidus	Moraceae	Bukit-Timah	BT-R03	KJ708839	BTSIN440-13
Artocarpus integer	Moraceae	Bukit-Timah	KJ594617	KJ708840	BTSIN466-13
Artocarpus kemando	Moraceae	Bukit-Timah	KJ594618	KJ708841	n/a
Artocarpus lowii	Moraceae	Bukit-Timah	KJ594620	KJ708843	BT-P01
Artocarpus scortechinii	Moraceae	Bukit-Timah	KJ594622	KJ708845	BTSIN212-13
Artocarpus styracifolius	Moraceae	Dinghushan	HQ415055	HQ415243	HQ415407
Asimina triloba	Annonaceae	SERC	L12631	GQ139722	GQ139861
Aspidosperma spruceanum	Apocynaceae	BCI	GQ981672	GQ981941	GQ982153
Astrocaryum standleyanum	Arecaceae	BCI	GQ981673	GQ981942	GQ982154
Astronium graveolens	Anacardiaceae	BCI	GQ981674	JQ586470	GQ982155
Attalea rostrata	Arecaceae	BCI	GQ981675	GQ981943	GQ982156
Aucuba chinensis	Garryaceae	Lienhuachih	KJ688006	KJ687468	KJ686681
Baccaurea macrocarpa	Phyllanthaceae	Bukit-Timah	KJ594624	KJ708847	BTSIN437-13
Bactris barronis	Arecaceae	BCI	GQ981676	GQ981944	GQ982157
Bactris coloniata	Arecaceae	BCI	GQ981677	n/a	GQ982158
Bactris major	Arecaceae	BCI	GQ981678	GQ981945	GQ982159
Beilschmiedia erythrophloia	Lauraceae	Lienhuachih	KJ688008	n/a	KJ686684
Beilschmiedia kunstleri	Lauraceae	Bukit-Timah	BT-R04	KJ708848	BT-P02
Beilschmiedia pendula	Lauraceae	Luquillo	GQ981679	EU153826	EU153945
Betula alleghaniensis	Betulaceae	Wabikon Lake	KJ593243	KJ592868	EU750442
Betula costata	Betulaceae	Changbaishan	CANGB012-14	n/a	n/a
Betula ermanii	Betulaceae	Changbaishan	CANGB013-14	n/a	n/a
Betula papyrifera	Betulaceae	Wabikon Lake	KJ593244	KJ592869	FJ011880
Betula platyphylla	Betulaceae	Changbaishan	CANGB014-14	CANGB014-15	CANGB014-16
Bhesa robusta	Celastraceae	Bukit-Timah	KJ594626	KJ708849	BT-P03
Bischofia javanica	Phyllanthaceae	Lienhuachih	AY663571	AY552420	n/a

Blastus cochinchinensis	Lamiaceae	Dinghushan/ Fushan	KJ688010	HQ415241	KJ686685
Borojoa panamensis	Rubiaceae	BCI	GQ981680	GQ981946	GQ982161
Brackenridgea hookeri	Ochnaceae	Bukit-Timah	KJ594629	n/a	BTSIN432-13
Bridelia retusa	Phyllanthaceae	Dinghushan	HQ415195	HQ415363	HQ415543
Bridelia tomentosa	Phyllanthaceae	Lienhuachih	KJ688016	n/a	n/a
Brosimum alicastrum	Moraceae	BCI	GQ981681	GQ981947	GQ982162
Brosimum guianense	Moraceae	BCI	GQ981682	GQ981948	GQ982163
Brunfelsia portoricensis	Solanaceae	Luquillo	HM446761	HM446659	HM446890
Buchenavia tetraphylla	Combretaceae	Luquillo	HM446762	HM446660	HM446891
Bunchosia nitida	Malpighiaceae	BCI	n/a	KJ012484	KJ426629
Byrsonima spicata	Malpighiaceae	Luquillo	HM446763	HM446661	HM446892
Byrsonima wadsworthii	Malpighiaceae	Luquillo	HM446764	HM446662	HM446893
Callicarpa bodinieri	Verbenaceae	Gutianshan	HQ427182	HQ427330	HQ427023
Callicarpa formosana	Verbenaceae	Lienhuachih	JQ618473	KP093632	JQ618420
Callicarpa giraldii	Verbenaceae	Gutianshan	HQ427184	HQ427332	HQ427025
Callicarpa kochiana	Verbenaceae	Fushan	KJ688022	KJ687476	KJ686695
Callicarpa rubella	Verbenaceae	Gutianshan	HQ427181	HQ427329	HQ427022
Callicarpa tikusikensis	Verbenaceae	Fushan	KJ688027	KJ687481	KJ686700
Calophyllum calaba	Clusiaceae	Luquillo	HM446765	n/a	HM446894
Calophyllum incrassatum	Clusiaceae	Bukit-Timah	KJ594633	n/a	BTSIN456-13
Calophyllum longifolium	Clusiaceae	BCI	GQ981683	HQ331555	GQ982164
Calophyllum macrocarpum	Clusiaceae	Bukit-Timah	KJ594637	n/a	n/a
Calophyllum membranaceum	Clusiaceae	Dinghushan	HQ415099	n/a	HQ415450
Calophyllum pulcherrimum	Clusiaceae	Bukit-Timah	KJ594635	n/a	n/a
Calophyllum teysmannii	Clusiaceae	Bukit-Timah	KJ594636	HQ331560	BTSIN197-13
Calophyllum wallichianum	Clusiaceae	Bukit-Timah	KJ594637	n/a	n/a

Calycogonium squamulosum	Melastomataceae	Luquillo	HM446766	n/a	HM446895
Camellia chekiang oleosa	Theaceae	Gutianshan	HQ427229	HQ427374	HQ427072
Camellia cuspidata	Theaceae	Gutianshan	HQ427225	HQ427370	HQ427068
Camellia fraterna	Theaceae	Gutianshan	HQ427224	n/a	n/a
Camellia furfuracea	Theaceae	Lienhuachih	KJ688031	KJ687485	KJ686704
Camellia nokoensis	Theaceae	Lienhuachih	KJ688034	KJ687486	KJ686705
Camellia salicifolia	Theaceae	Lienhuachih	KJ688037	n/a	KJ686707
Campnosperma auriculatum	Anacardiaceae	Bukit-Timah	KJ594639	KJ708854	BTSIN249-13
Canarium album	Burseraceae	Dinghushan	HQ415083	HQ415266	HQ415434
Canarium littorale	Burseraceae	Bukit-Timah	FJ466633	KJ708855	BTSIN606-13
Canarium pilosum	Burseraceae	Bukit-Timah	KJ594640	KJ708856	AY635378
Canarium tramdenum	Burseraceae	Dinghushan	HQ415084	HQ415267	HQ415435
Canthium confertum	Rubiaceae	Bukit-Timah	n/a	n/a	BTSIN144-13
Canthium glabrum	Rubiaceae	Bukit-Timah	KJ594641	n/a	BTSIN035-13
Canthium horridum	Rubiaceae	Dinghushan	HQ415226	HQ415390	HQ415572
Capparis frondosa	Capparaceae	BCI	GQ981684	GQ981949	GQ982165
Carallia brachiata	Rhizophoraceae	Dinghushan	HQ415233	HQ415397	HQ415579
Carpinus betulus	Betulaceae	Wytham	JN893226	JN895396	FJ011833
Carpinus caroliniana	Betulaceae	SERC	KJ593289	KJ592883	AY211430
Carpinus viminea	Betulaceae	Gutianshan	HQ427161	n/a	n/a
Carya cordiformis	Juglandaceae	SERC	KJ593294	KJ592887	HQ596633
Carya glabra	Juglandaceae	SERC	L12637	KF201333	KF201453
Carya laciniosa	Juglandaceae	SCBI	n/a	n/a	KF201451
Carya ovalis	Juglandaceae	SCBI	L12637	n/a	KF201453
Carya tomentosa	Juglandaceae	SERC	n/a	AF118039	KF201458
Caryota maxima	Arecaceae	Dinghushan	HQ415227	HQ415391	HQ415573
Caryota mitis	Arecaceae	Bukit-Timah	KJ594645	KJ708860	JF345055
Casearia aculeata	Salicaceae	BCI	GQ981685	GQ981950	GQ982166
Casearia arborea	Flacourtiaceae	Luquillo	GQ981686	HM446663	HM446896
Casearia commersoniana	Salicaceae	BCI	GQ981687	GQ981952	GQ982168
Casearia glomerata	Salicaceae	Dinghushan	HQ415115	HQ415293	HQ415465
Casearia guianensis	Salicaceae	BCI	HM446767	GQ981953	HM446897
Casearia membranacea	Flacourtiaceae	Fushan	KJ688042	KJ687490	KJ686709
Casearia sylvestris	Flacourtiaceae	Luquillo	HM446768	HM446664	HM446898
Casearia velutina	Salicaceae	Dinghushan	HQ415116	HQ415294	HQ415466

Cassipourea elliptica	Rhizophoraceae	BCI	GQ981690	GQ981955	GQ982171
Cassipourea guianensis	Rhizophoraceae	Luquillo	HM446769	HM446665	HM446899
Castanea dentata	Fagaceae	SCBI	KF613012	n/a	JQ677929
Castanea mollissima	Fagaceae	SERC	HQ336406	U92862	HQ336406
Castanea sativa	Fagaceae	Wytham	M94936	n/a	FN687510
Castanopsis carlesii	Fagaceae	Gutianshan	HQ427175	HQ427323	HQ427016
Castanopsis chinensis	Fagaceae	Dinghushan	HQ415235	HQ415399	HQ415581
Castanopsis cuspidata var. carlesii	Fagaceae	Fushan	KJ688046	KJ687491	KJ686711
Castanopsis eyrei	Fagaceae	Gutianshan	HQ427167	HQ427315	HQ427008
Castanopsis fargesii	Fagaceae	Gutianshan/ Lienhuachih	KJ688056	KJ687501	KJ686721
Castanopsis fissa	Fagaceae	Dinghushan	HQ415234	HQ415398	HQ415580
Castanopsis kawakamii	Fagaceae	Lienhuachih	KJ688057	KJ687503	KJ686723
Castanopsis lucida	Fagaceae	Bukit-Timah	KJ594649	KJ708864	BTSIN101-13
Castanopsis tibetana	Fagaceae	Gutianshan	HQ427172	HQ427320	HQ427013
Catunaregam spinosa	Rubiaceae	Dinghushan	HQ415178	HQ415349	HQ415527
Cavanillesia platanifolia	Malvaceae	BCI	GQ981691	GQ981956	GQ982172
Cecropia insignis	Urticaceae	BCI	GQ981692	JQ589383	GQ982173
Cecropia longipes	Urticaceae	BCI	GQ981693	GQ981957	GQ982174
Cecropia obtusifolia	Urticaceae	BCI	GQ981694	GQ981958	GQ982175
Cecropia schreberiana	Urticaceae	Luquillo	HM446770	HM446666	HM446900
Cedrela odorata	Meliaceae	BCI	GQ981695	GQ981959	GQ982176
Ceiba pentandra	Bombacaceae	Luquillo	GQ981696	GQ981960	GQ982177
Celtis biondii	Ulmaceae	Gutianshan	HQ427254	n/a	n/a
Celtis formosana	Cannabaceae	Lienhuachih	KJ688060	n/a	KJ686725
Celtis occidentalis	Cannabaceae	SERC	JX571800	n/a	n/a
Celtis schippii	Cannabaceae	BCI	GQ981697	GQ981961	GQ982178
Cespedesia spathulata	Ochnaceae	BCI	GQ981698	n/a	GQ982179
Cestrum macrophyllum	Solanaceae	Luquillo	HM446771	HM446667	HM446901

Cestrum megalophyllum	Solanaceae	BCI	JQ594113	n/a	GQ982180
Chamaedorea tepejilote	Arecaceae	BCI	GQ981699	GQ981962	GQ982181
Chamguava schippii	Myrtaceae	BCI	n/a	GQ981963	GQ982182
Chimarrhis parviflora	Rubiaceae	BCI	GQ981700	GQ981964	GQ982183
Chimonanthus salicifolius	Calycanthaceae	Gutianshan	HQ427177	HQ427325	HQ427018
Chionanthus domingensis	Oleaceae	Luquillo	HM446772	n/a	HM446902
Chionanthus virginicus	Oleaceae	SCBI	DQ006108	KJ772654	DQ006204
Chisocheton sarawakanus	Meliaceae	Bukit-Timah	KJ594652	KJ708867	BTSIN650-13
Chrysochlamys eclipes	Clusiaceae	BCI	GQ981702	HQ331570	GQ982184
Chrysophyllum argenteum	Sapotaceae	Luquillo	HM446773	HM446668	HM446904
Chrysophyllum cainito	Sapotaceae	BCI	GQ981701	GQ981966	GQ982186
Chrysophyllum lanceolatum	Sapotaceae	Dinghushan	HQ415117	HQ415295	HQ415467
Chrysophyllum lanceolatum var. stellatocarpon	Sapotaceae	Dinghushan	HQ415117	HQ415295	HQ415467
Cinnamomum austrosinense	Lauraceae	Fushan	KJ688061	n/a	KJ686727
Cinnamomum camphora	Lauraceae	Lienhuachih	KJ688064	KJ510888	KJ686728
Cinnamomum chekiangense	Lauraceae	Gutianshan	HQ427267	HQ427409	HQ427110
Cinnamomum elongatum	Lauraceae	Luquillo	HM446774	HM446669	HM446905
Cinnamomum micranthum	Lauraceae	Fushan	KJ688068	KJ687507	KJ686732
Cinnamomum montanum	Lauraceae	Luquillo	HM446775	HM446670	HM446906
Cinnamomum osmophloeum	Lauraceae	Lienhuachih	KJ688073	KJ687512	KJ686737
Cinnamomum sintoc	Lauraceae	Bukit-Timah	KJ594654	n/a	BTSIN326-13
Cinnamomum subavenium	Lauraceae	Gutianshan	HQ427266	HQ427408	HQ427109

Cinnamomum triplinerve	Lauraceae	BCI	GQ981703	GQ981967	GQ982187
Citharexylum caudatum	Verbenaceae	Luquillo	HM446776	HM446671	HM446907
Citharexylum fruticosum	Verbenaceae	Luquillo	HM446777	HM446672	HM446908
Citrus paradisi	Rutaceae	Luquillo	HM446778	n/a	HM446909
Clerodendron trichotomum	Lamiaceae	Gutianshan	HQ427186	n/a	n/a
Clerodendrum cyrtophyllum	Lamiaceae	Lienhuachih	KJ688085	KJ687521	KJ686745
Clerodendrum fortunatum	Lamiaceae	Dinghushan	HQ415230	HQ415394	HQ415576
Clerodendrum japonicum	Lamiaceae	Dinghushan	HQ415231	HQ415395	HQ415577
Clerodendrum trichotomum	Lamiaceae	Lienhuachih	KJ688086	KJ687522	KJ686746
Cleyera japonica	Pentaphylacaceae	Fushan	KJ688091	KJ687527	KJ686751
Clibadium erosum	Asteraceae	Luquillo	HM446779	HM446673	HM446910
Clidemia dentata	Melastomataceae	BCI	GQ981704	n/a	GQ982188
Clidemia octona	Melastomataceae	BCI	GQ981705	n/a	GQ982189
Clidemia septuplinervia	Melastomataceae	BCI	GQ981706	GQ981968	GQ982190
Clusia rosea	Clusiaceae	Luquillo	HM446780	HQ331583	HM446911
Coccoloba coronata	Polygonaceae	BCI	GQ981707	GQ981969	GQ982191
Coccoloba diversifolia	Polygonaceae	Luquillo	HM446781	HM446674	HM446912
Coccoloba manzinellensis	Polygonaceae	BCI	GQ981708	GQ981970	GQ982192
Coccoloba pyrifolia	Polygonaceae	Luquillo	KJ082215	HM446675	HM446913
Coffea arabica	Rubiaceae	Luquillo	HM446782	HM446676	HM446914
Cojoba rufescens	Fabaceae	BCI	GQ981709	GQ981971	GQ982193
Colubrina glandulosa	Rhamnaceae	BCI	JQ593576	n/a	n/a
Comocladia glabra	Anacardiaceae	Luquillo	HM446783	HM446677	HM446915
Conostegia bracteata	Melastomataceae	BCI	GQ981710	n/a	GQ982194
Conostegia cinnamomea	Melastomataceae	BCI	GQ981711	n/a	GQ982195
Cordia alliodora	Boraginaceae	BCI	GQ981712	GQ981972	GQ982196
Cordia bicolor	Boraginaceae	BCI	GQ981713	GQ981973	GQ982197
Cordia borinquensis	Boraginaceae	Luquillo	HM446784	HM446678	HM446916

Cordia lasiocalyx	Boraginaceae	BCI	GQ981714	n/a	GQ982198
Cordia sulcata	Boraginaceae	Luquillo	n/a	HM446679	HM446917
Cornus alternifolia	Cornaceae	Wabikon Lake	KJ593329	KJ592907	JF321212
Cornus florida	Cornaceae	SERC	EU002276	EU002175	GQ998105
Corylopsis glandulifera	Hamamelidaceae	Gutianshan	HQ427165	n/a	n/a
Corylus avellana	Betulaceae	Wytham	AY263929	AY263916	FJ011848
Corylus cornuta	Betulaceae	Wabikon Lake	KJ593340	KJ592915	FJ011850
Corylus mandshurica	Betulaceae	Changbaishan	CANGB015-14	CANGB015-15	CANGB015-16
Coussarea curvigemmia	Rubiaceae	BCI	GQ981715	GQ981974	GQ982199
Coutarea hexandra	Rubiaceae	BCI	GQ981716	GQ981975	GQ982200
Craibiodendron scleranthum	Ericaceae	Dinghushan	HQ415118	HQ415296	HQ415468
Crataegus maximowiczii	Rosaceae	Changbaishan	CANGB016-14	CANGB016-15	CANGB016-16
Crataegus monogyna	Rosaceae	Wytham	KC251347	KC251105	HG764982
Cratoxylum arborescens	Clusiaceae	Bukit-Timah	KJ594656	HQ331586	BTSIN454-13
Cratoxylum cochinchinense	Clusiaceae	Dinghushan	HQ415110	HQ331587	HQ415460
Cratoxylum formosum	Clusiaceae	Bukit-Timah	KJ594660	FJ670022	BTSIN131-13
Croton billbergianus	Euphorbiaceae	BCI	GQ981717	JQ58744	GQ982201
Croton lachnocarpus	Euphorbiaceae	Dinghushan	HQ415051	HQ415239	HQ415403
Croton laevifolius	Euphorbiaceae	Bukit-Timah	KJ594661	KJ708871	BTSIN276-13
Croton poecilanthus	Euphorbiaceae	Luquillo	HM446785	HM446680	HM446917
Crypteronia griffithii	Crypteroniaceae	Bukit-Timah	KJ594662	KJ708872	BTSIN447-13
Cryptocarya chinensis	Lauraceae	Fushan	KJ688094	KJ687529	KJ686754
Cryptocarya concinna	Lauraceae	Nanjenshan	KJ688716	KJ687907	KJ687311
Cryptocarya ferrea	Lauraceae	Bukit-Timah	KJ594663	n/a	n/a
Cryptocarya nitens	Lauraceae	Bukit-Timah	BT-R05	KJ708873	BT-P04
Cryptocarya nitens	Lauraceae	Bukit-Timah	n/a	KJ708873	n/a

Cryptocarya rugulosa	Lauraceae	Bukit-Timah	KJ594663	n/a	BTSIN122-13
Cunninghamia lanceolata	Cupressaceae	Gutianshan	HQ427238	HQ427381	HQ427081
Cupania cinerea	Sapindaceae	BCI	GQ981718	GQ981976	GQ982202
Cupania latifolia	Sapindaceae	BCI	GQ981719	GQ981977	GQ982203
Cupania rufescens	Sapindaceae	BCI	GQ981720	GQ981978	GQ982204
Cupania seemannii	Sapindaceae	BCI	GQ981721	GQ981979	GQ982205
Cyathea arborea	Cyatheaceae	Luquillo	HM446786	n/a	HM446918
Cyathea borinquena	Cyatheaceae	Luquillo	HM446787	n/a	HM446919
Cyathea lepifera	Cyatheaceae	Fushan	KJ688102	n/a	KJ686762
Cyathea petiolata	Cyatheaceae	BCI	EF463164	n/a	n/a
Cyathea podophylla	Cyatheaceae	Fushan	KJ688103	n/a	KJ686763
Cyathea spinulosa	Cyatheaceae	Fushan	KJ688108	n/a	KJ686769
Cyathocalyx ramuliflorus	Annonaceae	Bukit-Timah	KJ594665	KJ708874	BTSIN246-13
Cyclobalanopsis gilva	Fagaceae	Fushan	KJ688109	KJ687534	KJ686770
Cyclobalanopsis glauca	Fagaceae	Gutianshan	AB060571	HQ427316	HQ427009
Cyclobalanopsis gracilis	Fagaceae	Gutianshan	HQ427176	n/a	n/a
Cyclobalanopsis longinux	Fagaceae	Fushan	KJ688116	KJ687541	KJ686777
Cyclobalanopsis myrsinifolia	Fagaceae	Gutianshan	HQ427170	HQ427317	HQ427010
Cyclobalanopsis pachyloma	Fagaceae	Lienhuachih	KJ688117	KJ687542	KJ686778
Cyclobalanopsis sessilifolia	Fagaceae	Fushan	KJ688121	n/a	n/a
Cyrilla racemiflora	Cyrillaceae	Luquillo	HM446788	HM446681	HM446920
Dacryodes costata	Burseraceae	Bukit-Timah	BT-R06	KJ708876	BTSIN618-13
Dacryodes excelsa	Burseraceae	Luquillo	HM446789	AY594465	HM446921
Dacryodes laxa	Burseraceae	Bukit-Timah	KJ594667	n/a	BTSIN080-13
Dacryodes nervosa	Burseraceae	Bukit-Timah	KJ594668	KJ708877	n/a
Dalbergia hupeana	Fabaceae:Papil.	Gutianshan	HQ427142	HQ427296	HQ426987
Daphniphyllum oldhamii	Daphniphyllaceae	Dinghushan	HQ427162	HQ427311	HQ427004
Daphnopsis philippiana	Thymelaeaceae	Luquillo	HM446790	HM446682	HM446922
Dehaasia cuneata	Lauraceae	Bukit-Timah	AB925315	AJ24716	BTSIN229-13
Dendrobenthamia japonica	Cornaceae	Gutianshan	HQ427237	HQ427380	n/a

Dendropanax arboreus	Araliaceae	Luquillo	HM446791	HM446683	HM446923
Dendropanax dentiger	Araliaceae	Gutianshan	HQ427251	HQ427394	HQ427094
Desmopsis panamensis	Annonaceae	BCI	GQ981723	GQ981981	GQ982207
Deutzia parviflora var. amurensis	Hydrangeaceae	Changbaishan	CANGB017-14	n/a	n/a
Dialium indum	Fabaceae	Bukit-Timah	KJ594669	n/a	BTSIN013-13
Dillenia excelsa	Dilleniaceae	Bukit-Timah	KJ594670	KJ708879	BTSIN287-13
Dillenia grandifolia	Dilleniaceae	Bukit-Timah	KJ594671	n/a	BTSIN207-13
Dillenia suffruticosa	Dilleniaceae	Bukit-Timah	FJ860354	KJ708881	BTSIN634-13
Dimocarpus longan	Sapindaceae	Dinghushan	HQ415124	HQ415302	HQ415474
Diospyros artanthifolia	Ebenaceae	BCI	GQ981724	GQ981982	GQ982208
Diospyros clavigera	Ebenaceae	Bukit-Timah	n/a	KJ708885	BTSIN630-13
Diospyros confusa	Ebenaceae	Bukit-Timah	KJ594674	KJ708887	BTSIN478-13
Diospyros confusa	Ebenaceae	Bukit-Timah	KJ594674	KJ708887	BTSIN478-13
Diospyros coriacea	Ebenaceae	Bukit-Timah	BT-R07	KJ708889	BT-P05
Diospyros eriantha	Ebenaceae	Lienhuachih	KJ688132	KJ687552	KJ686790
Diospyros glaucifolia	Ebenaceae	Gutianshan	HQ427239	HQ427382	HQ427082
Diospyros maingayi	Ebenaceae	Bukit-Timah	KJ594677	KJ708892	BTSIN032-13
Diospyros morrisiana	Ebenaceae	Fushan	KJ688138	HQ415354	KJ686794
Diospyros pilosanthera	Ebenaceae	Bukit-Timah	KJ594678	KJ708893	BTSIN405-13
Diospyros styraciformis	Ebenaceae	Bukit-Timah	KJ594680	KJ708898	BTSIN002-13
Diospyros venosa	Ebenaceae	Bukit-Timah	KJ594681	KJ708900	BT-P06
Diospyros virginiana	Ebenaceae	SCBI	EU980774	DQ924064	FJ238227
Diplospora dubia	Rubiaceae	Dinghushan	HQ427201	HQ427350	HQ415437
Dipterocarpus caudatus	Dipterocarpaceae	Bukit-Timah	KJ594683	KJ708904	BTSIN234-13
Dipterocarpus cf kunstleri	Dipterocarpaceae	Bukit-Timah	KJ594684	n/a	BTSIN337-13
Dipterocarpus cornutus	Dipterocarpaceae	Bukit-Timah	BT-R08	KJ708905	BTSIN046-13
Dipterocarpus sublamellatus	Dipterocarpaceae	Bukit-Timah	BT-R09	KJ708906	BTSIN579-13
Dipterocarpus tempehes	Dipterocarpaceae	Bukit-Timah	KJ594685	KJ708907	BTSIN051-13

Dipteryx oleifera	Fabaceae	BCI	GQ981725	GQ981983	GQ982209
Dirca palustris	Thymelaeaceae	Wabikon Lake	KJ593370	KJ592930	n/a
Distyliopsis dunnii	Hamamelidaceae	Lienhuachih	KJ688140	n/a	KJ686627
Distylium myricoides	Hamamelidaceae	Gutianshan	HQ427166	n/a	n/a
Ditta myricoides	Euphorbiaceae	Luquillo	HM446792	HM446684	HM446924
Dracaena maingayi	Liliaceae	Bukit-Timah	KJ594686	n/a	BTSIN016-13
Drypetes alba	Euphorbiaceae	Luquillo	HM446793	n/a	HM446925
Drypetes glauca	Euphorbiaceae	Luquillo	HM446794	n/a	HM446926
Drypetes longifolia	Putranjivaceae	Bukit-Timah	KJ594687	n/a	BTSIN270-13
Drypetes pendula	Putranjivaceae	Bukit-Timah	KJ594688	n/a	n/a
Durio griffithii	Malvaceae	Bukit-Timah	n/a	KJ708908	BTSIN435-13
Durio singaporensis	Malvaceae	Bukit-Timah	KJ594689	KJ708910	BT-P07
Dysoxylum cyrtobotryum	Meliaceae	Bukit-Timah	KJ594692	KJ708913	BTSIN426-13
Ehretia acuminata	Boraginaceae	Gutianshan	HQ427271	HQ427413	HQ427114
Ehretia longiflora	Boraginaceae	Fushan	KJ688142	KJ687554	KJ686796
Elaeagnus umbellata	Elaeagnaceae	SCBI	HM849968	HM851107	HQ596679
Elaeis oleifera	Arecaceae	BCI	GQ981726	HQ265568	GQ982210
Elaeocarpus chinensis	Elaeocarpaceae	Gutianshan	HQ427153	HQ427304	HQ426997
Elaeocarpus ferrugineus	Elaeocarpaceae	Bukit-Timah	KJ594695	n/a	BTSIN409-13
Elaeocarpus floribundus	Elaeocarpaceae	Bukit-Timah	KJ594696	KJ708916	BTSIN428-13
Elaeocarpus japonicus	Elaeocarpaceae	Fushan	KJ688145	KJ687556	KJ686799
Elaeocarpus nitentifolius	Elaeocarpaceae	Dinghushan	HQ415078	HQ415262	HQ415429
Elaeocarpus petiolatus	Elaeocarpaceae	Bukit-Timah	KJ594700	KJ708920	BTSIN414-13
Elaeocarpus polystachyus	Elaeocarpaceae	Bukit-Timah	n/a	KJ708921	n/a
Elaeocarpus sylvestris	Elaeocarpaceae	Dinghushan	HQ415081	HQ415265	HQ415432
Eleutherococcus senticosus	Araliaceae	Changbaishan	CANGB018-14	n/a	n/a
Ellipanthus tomentosus	Connaraceae	Bukit-Timah	KJ594701	KJ708922	BT-P08
Endiandra maingayi	Lauraceae	Bukit-Timah	BT-R10	KJ708924	BTSIN652-13

Enkianthus quinqueflorus	Ericaceae	Dinghushan	HQ415063	HQ415251	HQ415415
Enterolobium schomburgkii	Fabaceae	BCI	GQ981727	GQ981984	GQ982211
Eriobotrya deflexa	Rosaceae	Fushan	KJ688166	JQ391008	KJ686818
Erythrina costaricensis	Fabaceae	BCI	n/a	GQ981985	GQ982212
Erythrophleum fordii	Fabaceae:Papil.	Dinghushan	HQ415085	HQ415268	HQ415436
Erythroxylum macrophyllum	Erythroxylaceae	BCI	GQ981728	GQ981986	GQ982213
Erythroxylum panamense	Erythroxylaceae	BCI	GQ981729	GQ981987	GQ982214
Eugenia coloradoensis	Myrtaceae	BCI	GQ981730	GQ981988	GQ982215
Eugenia domingensis	Myrtaceae	Luquillo	HM446795	HM446685	HM446927
Eugenia eggersii	Myrtaceae	Luquillo	HM446796	HM446686	HM446928
Eugenia galalonensis	Myrtaceae	BCI	GQ981731	n/a	n/a
Eugenia nesiotica	Myrtaceae	BCI	GQ981732	GQ981989	GQ982216
Eugenia oerstediana	Myrtaceae	BCI	GQ981733	GQ981990	n/a
Eugenia stahlii	Myrtaceae	Luquillo	HM446797	HM446687	HM446929
Euonymus carnosus	Celastraceae	Gutianshan	HQ427246	HQ427389	HQ427089
Euonymus laxiflorus	Celastraceae	Lienhuachih	KJ688171	KJ687571	KJ686824
Euonymus myrianthus	Celastraceae	Gutianshan	HQ427245	HQ427388	HQ427088
Euonymus nitidus	Celastraceae	Dinghushan	HQ427248	HQ427391	HQ415545
Euonymus oblongifolius	Celastraceae	Gutianshan	HQ427248	HQ427391	n/a
Euonymus phellomana	Celastraceae	Changbaishan	CANGB019-14	n/a	n/a
Euonymus tashiroi	Celastraceae	Lienhuachih	KJ688172	KJ687572	KJ686825
Euonymus verrucosus	Celastraceae	Changbaishan	CANGB020-14	n/a	n/a
Eurya chinensis	Pentaphylacaceae	Dinghushan	HQ415122	HQ415300	HQ415472
Eurya loquaiana	Pentaphylacaceae	Fushan	KJ688176	KJ687578	KJ686830
Eurya macartneyi	Pentaphylacaceae	Dinghushan	HQ415121	HQ415299	HQ415471
Eurya muricata	Pentaphylacaceae	Gutianshan	HQ427228	HQ427373	HQ427071
Eurya rubiginosa	Pentaphylacaceae	Gutianshan	HQ427222	HQ427368	HQ427065
Eurycoma longifolia	Simaroubaceae	Bukit-Timah	KJ594704	KJ708926	BTSIN269-13
Euscaphis japonica	Staphyleaceae	Gutianshan	HQ427180	HQ427328	HQ427021
Eustigma balansae	Hamamelidaceae	Dinghushan	HQ415214	HQ415379	HQ415561

Eustigma oblongifolium	Hamamelidaceae	Lienhuachih	KJ688184	KJ687581	KJ686839
Evodia faugeaii	Rutaceae	Gutianshan	KF912881	n/a	n/a
Fagraea fragrans	Gentianaceae	Bukit-Timah	KJ594706	n/a	n/a
Fagus grandifolia	Fagaceae	SERC	KJ593410	KJ592948	HQ596698
Fagus sylvatica	Fagaceae	Wytham	JN641795	JN895059	FN687511
Faramea occidentalis	Rubiaceae	Luquillo	HM446798	HM446688	HM446930
Ficus ampelas	Moraceae	Lienhuachih	KJ688186	JQ773505	KJ686841
Ficus bullenei	Moraceae	BCI	GQ981735	GQ981991	GQ982218
Ficus citrifolia	Moraceae	Luquillo	HM446799	HM446689	HM446931
Ficus costaricana	Moraceae	BCI	GQ981737	GQ981993	GQ982220
Ficus crassinervia	Moraceae	Luquillo	HM446800	HM446690	HM446932
Ficus erecta	Moraceae	Gutianshan	HQ427220	n/a	n/a
Ficus erecta var. beecheyana	Moraceae	Fushan	KJ688187	KJ687582	KJ686842
Ficus fistulosa	Moraceae	Lienhuachih	KJ688191	n/a	KJ686845
Ficus formosana	Moraceae	Fushan	KJ688197	KJ687585	KJ686851
Ficus grossularioides	Moraceae	Bukit-Timah	KJ594707	KJ708927	n/a
Ficus insipida	Moraceae	BCI	GQ981738	GQ981994	GQ982221
Ficus lamponga	Moraceae	Bukit-Timah	n/a	n/a	BTSIN132-13
Ficus maxima	Moraceae	BCI	GQ981739	GQ981995	GQ982222
Ficus nervosa	Moraceae	Lienhuachih	KJ688206	KJ687592	KJ686860
Ficus pandurata	Moraceae	Dinghushan	HQ415153	HQ415327	HQ415503
Ficus popenoei	Moraceae	BCI	GQ981741	GQ981997	GQ982224
Ficus scortechinii	Moraceae	Bukit-Timah	n/a	KJ708928	n/a
Ficus sintenisii	Moraceae	Luquillo	HM446801	HM446691	HM446933
Ficus sinuata	Moraceae	Bukit-Timah	KJ594709	KJ708929	BTSIN461-13
Ficus superba	Moraceae	Dinghushan	HQ415150	HQ415324	HQ415500
Ficus superba var. japonica	Moraceae	Fushan	KJ688208	n/a	n/a
Ficus tonduzii	Moraceae	BCI	GQ981742	GQ981998	GQ982225
Ficus trigonata	Moraceae	BCI	GQ981743	n/a	GQ982226
Ficus variegata	Moraceae	Dinghushan	HQ415154	n/a	n/a
Ficus yoponensis	Moraceae	BCI	GQ981744	GQ981999	GQ982227
Flacourtia rukam	Salicaceae	Bukit-Timah	n/a	JF738593	BTSIN626-13
Flueggea virosa	Phyllanthaceae	Dinghushan	HQ415223	HQ415387	HQ415569
Fraxinus americana	Oleaceae	Wabikon Lake	KJ593422	KJ592959	HQ596701
Fraxinus excelsior	Oleaceae	Wytham	FJ862056	FJ395414	FJ395513
Fraxinus insularis	Oleaceae	Gutianshan	HQ427187	HQ427335	HQ427029

Fraxinus mandshurica	Oleaceae	Changbaishan	CANGB021-14	CANGB021-15	CANGB021-16
Fraxinus nigra	Oleaceae	Wabikon Lake	KJ593423	KJ592960	HQ596702
Fraxinus pennsylvanica	Oleaceae	SERC	HQ590104	HQ593301	HQ596703
Gaertnera grisea	Rubiaceae	Bukit-Timah	n/a	AM117227	BTSIN639-13
Galearia fulva	Pandaceae	Bukit-Timah	KJ594713	KJ708930	BT-P09
Galearia maingayi	Pandaceae	Bukit-Timah	KJ594714	KJ708931	n/a
Garcinia atroviridis	Clusiaceae	Bukit-Timah	KJ594716	n/a	BTSIN004-13
Garcinia eugeniifolia	Clusiaceae	Bukit-Timah	KJ594720	n/a	n/a
Garcinia forbesii	Clusiaceae	Bukit-Timah	KJ594717	n/a	BTSIN418-13
Garcinia hombroniana	Clusiaceae	Bukit-Timah	KJ594718	KJ708932	BTSIN295-13
Garcinia intermedia	Clusiaceae	BCI	GQ981745	HQ331600	GQ982228
Garcinia madruno	Clusiaceae	BCI	GQ981746	JQ587259	GQ982229
Garcinia multiflora	Clusiaceae	Nanjenshan	HQ415192	n/a	HQ415541
Garcinia oblongifolia	Clusiaceae	Dinghushan	HQ415193	n/a	n/a
Garcinia scortechinii	Clusiaceae	Bukit-Timah	KJ594720	HQ331607	BTSIN381-13
Gardenia jasminoides	Rubiaceae	Lienhuachih	KJ688214	KJ687596	KJ686866
Gardenia tubifera	Rubiaceae	Bukit-Timah	KJ594722	KJ708933	BTSIN449-13
Genipa americana	Rubiaceae	Luquillo	HM446802	HM446692	HM446934
Glochidion acuminatum	Phyllanthaceae	Fushan	KJ688215	KJ687598	KJ686871
Glochidion hypoleucum	Phyllanthaceae	Bukit-Timah	KJ594724	n/a	BTSIN174-13
Glochidion puberulum	Phyllanthaceae	Gutianshan	HQ427128	HQ427285	HQ426975
Glochidion wrightii	Phyllanthaceae	Dinghushan	HQ415187	HQ415357	HQ415536
Gluta wallichii	Anacardiaceae	Bukit-Timah	KJ594726	KJ708937	BTSIN201-13
Glycosmis citrifolia	Rutaceae	Lienhuachih	KJ688225	KJ687602	KJ686875
Glycosmis parviflora	Rutaceae	Dinghushan	HQ415179	HQ415350	HQ415528
Gnetum gnemon	Gnetaceae	Bukit-Timah	AY296534	n/a	n/a
Gomphandra quadrifida	Stemonuraceae	Bukit-Timah	KJ594729	KJ708940	BTSIN346-13
Gomphia serrata	Ochnaceae	Bukit-Timah	KJ594730	AB233803	BTSIN015-13
Goniothalamus macrophyllus	Annonaceae	Bukit-Timah	BT-R11	KJ708941	BTSIN267-13

Goniothalamus tapis	Annonaceae	Bukit-Timah	n/a	KJ708941	n/a
Gonystylus confusus	Thymelaeaceae	Bukit-Timah	KJ594731	KJ708943	BTSIN357-13
Gonzalagunia spicata	Rubiaceae	Luquillo	HM446803	HM446693	HM446935
Gordonia axillaris	Theaceae	Fushan	KJ688226	n/a	KJ686876
Gordonia singaporeana	Theaceae	Bukit-Timah	KJ594733	KJ708945	BTSIN142-13
Guapira standleyana	Nyctaginaceae	BCI	GQ981748	GQ982001	GQ982231
Guarea bullata	Meliaceae	BCI	JQ592719	JQ588338	n/a
Guarea fuzzy	Meliaceae	BCI	GQ981749	n/a	GQ982232
Guarea glabra	Meliaceae	Luquillo	HM446804	HM446694	HM446936
Guarea grandifolia	Meliaceae	BCI	GQ981750	GQ982002	GQ982233
Guarea guidonia	Meliaceae	Luquillo	HM446805	HM446695	HM446937
Guatteria caribaea	Annonaceae	Luquillo	HM446806	HM446696	HM446938
Guatteria dumetorum	Annonaceae	BCI	GQ981752	n/a	GQ982235
Guazuma ulmifolia	Sterculiaceae	Luquillo	HM446807	GQ982003	HM446939
Guettarda foliacea	Rubiaceae	BCI	GQ981754	GQ982004	GQ982237
Guettarda valenzuelana	Rubiaceae	Luquillo	HM446808	HM446697	HM446940
Guioa pubescens	Sapindaceae	Bukit-Timah	KJ594735	KJ708947	BTSIN411-13
Gymnacranthera farquhariana	Myristicaceae	Bukit-Timah	KJ594739	KJ708949	BTSIN482-13
Gynotroches axillaris	Rhizophoraceae	Bukit-Timah	KJ594741	n/a	n/a
Hamamelis virginiana	Hamamelidaceae	SCBI	DQ352368	KJ840926	EU595863
Hamelia axillaris	Rubiaceae	Luquillo	HM446809	GQ982006	HM446941
Hamelia patens	Rubiaceae	BCI	GQ981757	GQ982007	GQ982240
Hampea appendiculata	Malvaceae	BCI	GQ981758	JQ588226	GQ982241
Hasseltia floribunda	Salicaceae	BCI	GQ981759	GQ982008	GQ982242
Heisteria acuminata	Olacaceae	BCI	GQ981760	GQ982009	GQ982243
Heisteria concinna	Olacaceae	BCI	GQ981761	GQ982010	GQ982244
Heisteria scandens	Olacaceae	BCI	JQ593018	JQ588534	BCI-P01
Helicia cochinchinensis	Proteaceae	Lienhuachih	KJ688230	HQ415347	KJ686880
Helicia formosana	Proteaceae	Fushan	n/a	KJ687603	n/a
Helicia rengetiensis	Proteaceae	Lienhuachih	KJ688232	KJ687605	KJ686881
Henriettea fascicularis	Melastomataceae	Luquillo	HM446810	HM446698	HM446941
Heritiera borneensis	Malvaceae	Bukit-Timah	KJ594742	KJ708951	BTSIN423-13

Heritiera elata	Malvaceae	Bukit-Timah	KJ594744	KJ708952	BTSIN176-13
Herrania purpurea	Malvaceae	BCI	GQ981762	GQ982011	GQ982245
Hibiscus pernambucensis	Malvaceae	Luquillo	HM446811	HM446699	HM446943
Hieronyma alchorneoides	Euphorbiaceae	BCI	JQ626093	GQ982012	GQ982246
Hirtella americana	Chrysobalanaceae	BCI	GQ981763	n/a	GQ982247
Hirtella rugosa	Chrysobalanaceae	Luquillo	HM446812	n/a	HM446944
Hirtella triandra	Chrysobalanaceae	BCI	GQ981764	JQ587241	GQ982248
Homalium cochinchinense	Salicaceae	Dinghushan	HQ415194	HQ415362	HQ415542
Homalium cochinchinensis	Salicaceae	Lienhuachih	KJ688234	KJ687606	KJ686883
Homalium racemosum	Flacourtiaceae	Luquillo	HM446813	HM446700	HM446945
Hopea ferrugineum	Dipterocarpaceae	Bukit-Timah	KJ594745	n/a	n/a
Hopea griffithii	Dipterocarpaceae	Bukit-Timah	n/a	KJ708954	BTSIN625-13
Hopea sangal	Dipterocarpaceae	Bukit-Timah	KJ594747	KJ708957	BTSIN058-13
Hovenia acerba	Rhamnaceae	Dinghushan	HQ415232	HQ415396	HQ415578
Hovenia trichocarpa	Rhamnaceae	Gutianshan	HQ427241	HQ427384	HQ427084
Hura crepitans	Euphorbiaceae	BCI	GQ981765	GQ982013	GQ982249
Hybanthus prunifolius	Violaceae	BCI	GQ981766	GQ982014	GQ982250
Hydrangea chinensis	Hydrangeaceae	Fushan	KJ688235	n/a	KJ686884
Idesia polycarpa	Salicaceae	Gutianshan	HQ427272	HQ427414	HQ427115
Idesia polycarpa	Salicaceae	Gutianshan	HQ427272	HQ427414	n/a
Ilex aquifolium	Aquifoliaceae	Wytham	FJ395601	FJ395435	FN675791
Ilex asprella	Aquifoliaceae	Lienhuachih	KJ688236	KJ687607	KJ686885
Ilex chapaensis	Aquifoliaceae	Dinghushan	HQ415069	HQ415254	HQ415421
Ilex chinensis	Aquifoliaceae	Gutianshan	JF941992	JF954086	KF255753
Ilex cochinchinensis	Aquifoliaceae	Dinghushan	HQ415071	HQ415256	HQ415423
Ilex elmerrilliana	Aquifoliaceae	Gutianshan	HQ427132	n/a	n/a
Ilex ficoidea	Aquifoliaceae	Fushan	KJ688237	KJ687608	KJ686886
Ilex formosana	Aquifoliaceae	Fushan	KJ688248	KJ687619	KJ686897
Ilex goshiensis	Aquifoliaceae	Fushan	KJ688253	KJ687624	KJ686902
Ilex hayataiana	Aquifoliaceae	Fushan	KJ688262	KJ687633	KJ686911
Ilex latifolia	Aquifoliaceae	Gutianshan	HQ427134	HQ427289	HQ426980
Ilex lonicerifolia	Aquifoliaceae	Fushan	KJ688263	KJ687634	KJ686912
Ilex macrocarpa	Aquifoliaceae	Dinghushan	HQ415064	n/a	HQ415416
Ilex memecylifolia	Aquifoliaceae	Dinghushan	HQ415065	HQ415252	HQ415417

Ilex micrococca	Aquifoliaceae	Gutianshan	HQ427135	HQ427290	HQ426981
Ilex opaca	Aquifoliaceae	SERC	EF590536	GQ248140	GQ248321
Ilex pubescens	Aquifoliaceae	Fushan	KJ688275	KJ687646	KJ686924
Ilex rotunda	Aquifoliaceae	Dinghushan	HQ427138	HQ415255	HQ415422
Ilex sideroxyloides	Aquifoliaceae	Luquillo	L01928	n/a	n/a
Ilex suaveolens	Aquifoliaceae	Gutianshan	HQ427139	HQ427293	HQ426984
Ilex triflora	Aquifoliaceae	Dinghushan	HQ415068	n/a	HQ415420
Ilex verticillata	Aquifoliaceae	Wabikon Lake	KJ593485	KJ592999	EU359326
Ilex wilsonii	Aquifoliaceae	Gutianshan	HQ427140	HQ427294	HQ426985
Illicium lanceolatum	Schisandraceae	Gutianshan	HQ427126	HQ427283	HQ426973
Indocalamus longiauritus	Poaceae	Dinghushan	HQ415166	HQ415338	HQ415515
Inga acuminata	Fabaceae	BCI	GQ982015	GQ981767	GQ982251
Inga cocleensis	Fabaceae	BCI	GQ981768	GQ982016	GQ982252
Inga goldmanii	Fabaceae	BCI	GQ981769	GQ982017	GQ982253
Inga laurina	Fabaceae	BCI	HM446814	HM446701	HM446946
Inga marginata	Fabaceae	BCI	GQ981771	GQ982018	GQ982255
Inga nobilis	Fabaceae	BCI	GQ981772	GQ982019	GQ982256
Inga oerstediana	Fabaceae	BCI	GQ981773	JQ587681	GQ982257
Inga pezizifera	Fabaceae	BCI	GQ981774	GQ982020	GQ982258
Inga punctata	Fabaceae	BCI	GQ981775	GQ982021	GQ982259
Inga ruiziana	Fabaceae	BCI	GQ981776	GQ982022	GQ982260
Inga sapindoides	Fabaceae	BCI	GQ981777	GQ982023	GQ982261
Inga spectabilis	Fabaceae	BCI	GQ981778	GQ982024	GQ982262
Inga thibaudiana	Fabaceae	BCI	GQ981779	GQ982025	GQ982263
Inga umbellifera	Fabaceae	BCI	GQ981780	GQ982026	GQ982264
Inga vera	Fabaceae:Mimos.	Luquillo	HM446815	HM446702	HM446947
Irvingia malayana	Irvingiaceae	Bukit-Timah	KJ594753	KJ708962	n/a
Itea chinensis	Iteaceae	Dinghushan	HQ415186	HQ415356	HQ415535
Itea oblonga	Iteaceae	Gutianshan	HQ427159	n/a	n/a
Itea parviflora	Iteaceae	Fushan	KJ688283	n/a	n/a
Ixonanthes reticulata	Ixonanthaceae	Bukit-Timah	AB233893	AB233789	BT-P10
Ixora chinensis	Rubiaceae	Dinghushan	HQ415123	HQ415301	HQ415473
Ixora ferrea	Rubiaceae	Luquillo	HM446816	HM446703	HM446948
Ixora javanica	Rubiaceae	Bukit-Timah	KJ594754	KJ708963	BTSIN271-13
Jacaranda copaia	Bignoniaceae	BCI	GQ981781	n/a	GQ982265
Juglans cinerea	Juglandaceae	Wabikon Lake	HQ590142	HQ594030	HQ596735
Juglans mandshurica	Juglandaceae	Changbaishan	CANGB022-14	CANGB022-15	CANGB022-16

Juglans nigra	Juglandaceae	SCBI	U00437	U92851	HQ596736
Knema cinerea	Myristicaceae	Bukit-Timah	KJ594758	KJ708967	BTSIN199-13
Knema hookeriana	Myristicaceae	Bukit-Timah	KJ594759	KJ708968	BTSIN417-13
Knema laurina	Myristicaceae	Bukit-Timah	KJ594761	KJ708970	BTSIN318-13
Knema patentinervia	Myristicaceae	Bukit-Timah	KJ594762	KJ708971	BTSIN597-13
Koilodepas longifolium	Euphorbiaceae	Bukit-Timah	KJ594763	n/a	BTSIN362-13
Kokoona reflexa	Celastraceae	Bukit-Timah	KJ594764	KJ708972	BTSIN075-13
Koompassia malaccensis	Fabaceae	Bukit-Timah	KJ594765	KJ708973	BTSIN293-13
Lacistema aggregatum	Lacistemataceae	BCI	n/a	GQ982027	n/a
Lacmellea panamensis	Apocynaceae	BCI	GQ981782	GQ982028	GQ982266
Laetia procera	Salicaceae	BCI	HM446817	HM446704	HM446949
Laetia thamnia	Salicaceae	BCI	GQ981784	GQ982029	GQ982268
Lafoensia punicifolia	Lythraceae	BCI	BCI-R01	GQ982030	GQ982269
Lagerstroemia subcostata	Lythraceae	Fushan	KJ688287	n/a	KJ686933
Larix decidua	Pinaceae	Wytham	NC_016058	NC_016058	NC_016058
Larix gmelinii	Pinaceae	Changbaishan	CANGB023-14	CANGB023-15	CANGB023-16
Larix laricina	Pinaceae	Wabikon Lake	KJ593496	n/a	n/a
Lasianthus appressihirtus var. maximus	Rubiaceae	Fushan	KJ688296	KJ687656	KJ686941
Lasianthus bunzanensis	Rubiaceae	Lienhuachih	KJ688297	n/a	KJ686942
Lasianthus curtisii	Rubiaceae	Fushan	KJ688298	KJ687657	KJ686943
Lasianthus cyanocarpus	Rubiaceae	Lienhuachih	KJ688307	KJ687666	KJ686952
Lasianthus fordii	Rubiaceae	Fushan	KJ688311	KJ687670	KJ686956
Lasianthus japonicus	Rubiaceae	Gutianshan	HQ427196	HQ427345	HQ427039
Lasianthus microstachys	Rubiaceae	Fushan	KJ688316	KJ687675	KJ686961
Lasianthus obliquinervis	Rubiaceae	Lienhuachih	KJ688325	KJ687683	KJ686970
Lasianthus wallichii	Rubiaceae	Nanjenshan	KJ688779	KJ687686	KJ687370
Lepisanthes	Sapindaceae	Bukit-Timah	KJ594767	KJ708974	BTSIN205-13

fruticosa					
Lespedeza formosa	Fabaceae	Gutianshan	HQ427143	n/a	n/a
Licania hypoleuca	Chrysobalanaceae	BCI	GQ981786	GQ982032	GQ982271
Licania platypus	Chrysobalanaceae	BCI	GQ981787	GQ982033	GQ982272
Licania splendens	Chrysobalanaceae	Bukit-Timah	KJ594768	KJ708975	BTSIN474-13
Limlia uraiana	Fagaceae	Fushan	KJ688332	KJ687690	KJ686979
Lindackeria laurina	Achariaceae	BCI	GQ981788	GQ982034	GQ982273
Lindera aggregata	Lauraceae	Gutianshan	HQ427260	HQ427402	HQ427103
Lindera benzoin	Lauraceae	SERC	AY337732	AJ247169	AF268788
Lindera chunii	Lauraceae	Dinghushan	HQ415171	HQ415342	HQ415520
Lindera communis	Lauraceae	Fushan	KJ688337	KJ687695	KJ686984
Lindera glauca	Lauraceae	Gutianshan	HQ427265	HQ427407	n/a
Lindera metcalfiana	Lauraceae	Dinghushan	HQ415172	HQ415343	HQ415521
Lindera reflexa	Lauraceae	Gutianshan	HQ427264	HQ427406	HQ427107
Liquidambar formosana	Altingiaceae	Gutianshan	HQ427164	HQ427313	HQ427006
Liquidambar styraciflua	Altingiaceae	SERC	AF119181	AF133218	AB445366
Liriodendron tulipifera	Magnoliaceae	SERC	AY008947	AF123480	AB021047
Litchi chinensis	Sapindaceae	Dinghushan	HQ415120	HQ415298	HQ415470
Lithocarpus amygdalifolius	Fagaceae	Lienhuachih	KJ688347	KJ687704	KJ686993
Lithocarpus glaber	Fagaceae	Gutianshan	HQ427174	HQ427322	HQ427015
Lithocarpus gracilis	Fagaceae	Bukit-Timah	n/a	KJ708976	n/a
Litsea accedens	Lauraceae	Bukit-Timah	BT-R12	KJ708978	BTSIN660-13
Litsea acuminata	Lauraceae	Fushan	KJ688350	KJ687706	KJ686996
Litsea castanea	Lauraceae	Bukit-Timah	BT-R13	KJ708981	BTSIN390-13
Litsea coreana	Lauraceae	Gutianshan	HQ427263	HQ427405	HQ427106
Litsea costalis	Lauraceae	Bukit-Timah	KJ594769	KJ708982	BTSIN073-13
Litsea cubeba	Lauraceae	Lienhuachih	KJ688359	KJ687710	KJ687004
Litsea elliptica	Lauraceae	Bukit-Timah	KJ594770	KJ708983	BTSIN244-13
Litsea elongata	Lauraceae	Gutianshan	HQ427261	HQ427403	HQ427104
Litsea firma	Lauraceae	Bukit-Timah	KJ594773	n/a	n/a
Litsea grandis	Lauraceae	Bukit-Timah	KJ594774	KJ708986	BT-P11
Litsea hypophaea	Lauraceae	Lienhuachih	KJ688361	KJ687711	KJ687006
Litsea ridleyi	Lauraceae	Bukit-Timah	KJ594775	KJ708987	BTSIN204-13
Litsea rotundifolia	Lauraceae	Dinghushan	HQ415128	HQ415306	HQ415478
Litsea verticillata	Lauraceae	Dinghushan	HQ415129	n/a	HQ415479
Lonchocarpus heptaphyllus	Fabaceae	BCI	GQ981789	GQ982035	GQ982274

Lonchocarpus latifolius	Fabaceae:Papil.	Luquillo	HM446818	HM446705	HM446950
Lonicera praeflorens	Caprifoliaceae	Changbaishan	CANGB024-14	n/a	n/a
Lophopetalum pallidum	Celastraceae	Bukit-Timah	KJ594776	n/a	BTSIN068-13
Loropetalum chinensis	Hamamelidaceae	Gutianshan	HQ427163	n/a	n/a
Lozania pittieri	Lacistemataceae	BCI	GQ981790	n/a	GQ982275
Ludwigia octovalvis	Onagraceae	Luquillo	L1022	n/a	n/a
Luehea seemannii	Malvaceae	BCI	GQ981791	GQ982036	GQ982276
Lyonia ovalifolia	Ericaceae	Gutianshan	HQ427150	HQ427302	HQ426994
Maackia amurensis	Fabaceae	Changbaishan	CANGB025-14	n/a	n/a
Macaranga andamanica	Euphorbiaceae	Dinghushan	HQ415129	HQ415380	HQ415479
Macaranga bancana	Euphorbiaceae	Bukit-Timah	KJ594777	n/a	n/a
Macaranga lowii	Euphorbiaceae	Bukit-Timah	KJ594780	n/a	n/a
Machilus breviflora	Lauraceae	Dinghushan	HQ415159	HQ415332	HQ415508
Machilus chinensis	Lauraceae	Dinghushan	HQ415162	HQ415335	HQ415511
Machilus grijsii	Lauraceae	Dinghushan	KF569893	KF740400	KP095575
Machilus japonica var. kusanoi	Lauraceae	Fushan	KJ688368	KJ687713	KJ687012
Machilus kwangtungensis	Lauraceae	Dinghushan	HQ415161	HQ415334	HQ415510
Machilus pauhoi	Lauraceae	Gutianshan	HQ427276	HQ427418	HQ427119
Machilus phoenicis	Lauraceae	Dinghushan	HQ415160	HQ415333	HQ415509
Machilus robusta	Lauraceae	Dinghushan	HQ415164	HQ415336	HQ415513
Machilus thunbergii	Lauraceae	Fushan	KJ688372	KJ687715	KJ687017
Machilus velutina	Lauraceae	Dinghushan	HQ415165	HQ415337	HQ415514
Machilus zuihoensis	Lauraceae	Fushan	KJ688384	KJ687717	KJ687031
Maclura cochinchinensis	Moraceae	Gutianshan	HQ427219	HQ427365	HQ427062
Maclurodendron porteri	Rutaceae	Bukit-Timah	KJ594781	n/a	n/a
Macrocnemum roseum	Rubiaceae	BCI	GQ981792	GQ982037	GQ982278
Madhuca malaccensis	Sapotaceae	Bukit-Timah	KJ594784	KJ708991	BTSIN041-13
Maesa japonica	Primulaceae	Fushan	KJ688389	KJ687721	KJ687035
Maesa perlaria var. formosana	Primulaceae	Fushan	KJ688390	KJ687722	KJ687036
Maesa ramentacea	Primulaceae	Bukit-Timah	n/a	KJ708993	BTSIN328-13

Maesa salicifolia	Primulaceae	Dinghushan	HQ415072	HQ415257	HQ415424
Magnolia cylindrica	Magnoliaceae	Gutianshan	HQ427278	HQ427420	HQ427121
Magnolia paenetalauma	Magnoliaceae	Dinghushan	HQ415131	HQ415308	HQ415481
Magnolia splendens	Magnoliaceae	Luquillo	HM446819	KJ012663	HM446951
Mahonia bealei	Berberidaceae	Gutianshan	L75871	n/a	n/a
Mallotus apelta	Euphorbiaceae	Dinghushan	HQ427129	HQ427286	HQ415565
Mallotus japonicus	Euphorbiaceae	Fushan	KJ688398	AB268027	KJ686635
Mallotus paniculatus	Euphorbiaceae	Fushan	KJ688404	HQ415384	KJ686630
Mallotus penangensis	Euphorbiaceae	Bukit-Timah	KJ594787	n/a	n/a
Mallotus philippensis	Euphorbiaceae	Lienhuachih	KJ688410	HQ415385	KJ686636
Malpighia romeroana	Malpighiaceae	BCI	GQ981793	GQ982038	GQ982279
Malus baccata	Rosaceae	Changbaishan	CANGB026-14	CANGB026-15	CANGB026-16
Malus leiocalyca	Rosaceae	Gutianshan	HQ427202	HQ427351	HQ427045
Malus pumila	Rosaceae	Wabikon Lake	KJ593530	KJ593022	n/a
Mangifera indica	Anacardiaceae	Luquillo	HM446821	JQ586473	HM446953
Manglietia yuyuanensis	Magnoliaceae	Gutianshan	HQ427274	n/a	n/a
Manilkara bidentata	Sapotaceae	Luquillo	HM446822	HM446708	HM446954
Maquira guianensis	Moraceae	BCI	GQ981794	GQ982039	GQ982280
Margaritaria nobilis	Phyllanthaceae	BCI	HM446823	HM446709	HM446955
Marila laxiflora	Calophyllaceae	BCI	GQ981796	n/a	GQ982282
Matayba domingensis	Sapindaceae	Luquillo	HM446824	HM446710	HM446956
Matthaea sancta	Monimiaceae	Bukit-Timah	KJ594794	KJ708998	BTSIN442-13
Maytenus elongata	Celastraceae	Luquillo	HM446825	HM446711	HM446957
Maytenus schippii	Celastraceae	BCI	GQ981797	GQ982041	GQ982283
Meiogyne virgata	Annonaceae	Bukit-Timah	KJ594795	KJ708999	JX544784
Melastoma candidum	Melastomataceae	Fushan	KJ688413	KP093302	KJ687041
Melastoma sanguineum	Melastomataceae	Dinghushan	HQ415218	n/a	HQ415564
Melicope glabra	Rutaceae	Bukit-Timah	KJ594799	KJ709002	BTSIN431-13
Melicope pteleifolia	Rutaceae	Lienhuachih	KJ688417	KJ510924	KJ687045
Meliosma flexuosa	Sabiaceae	Gutianshan	HQ427214	HQ427361	HQ427057

Meliosma fordii	Sabiaceae	Dinghushan	HQ415133	n/a	HQ415483
Meliosma herbertii	Sabiaceae	Luquillo	HM446826	HM446712	HM446958
Meliosma lanceolata	Sabiaceae	Bukit-Timah	KJ594801	KJ709003	n/a
Meliosma oldhamii	Sabiaceae	Gutianshan	HQ427213	HQ427360	HQ427056
Meliosma rhoifolia	Sabiaceae	Lienhuachih	KJ688420	n/a	n/a
Meliosma rigida	Sabiaceae	Dinghushan	HQ415132	HQ415309	HQ415482
Meliosma squamulata	Sabiaceae	Fushan	KJ688423	n/a	KJ687049
Memecylon ligustrifolium	Melastomataceae	Dinghushan	HQ415087	HQ415270	HQ415438
Memecylon megacarpum	Melastomataceae	Bukit-Timah	KJ594802	n/a	BTSIN151-13
Memecylon minutiflorum	Melastomataceae	Bukit-Timah	KJ594803	n/a	n/a
Memecylon nigrescens	Melastomataceae	Dinghushan	HQ415088	HQ415271	HQ415439
Mesechites trifidus	Apocynaceae	BCI	AJ419749	EF456269	BCI-P02
Mezzettia parviflora	Annonaceae	Bukit-Timah	KJ594804	KJ709004	BTSIN184-13
Michelia compressa	Magnoliaceae	Fushan	KJ688428	KJ687727	KJ687054
Michelia foveolata	Magnoliaceae	Dinghushan	HQ415092	HQ415275	HQ415443
Michelia maudiae	Magnoliaceae	Dinghushan	HQ415093	HQ415276	HQ415444
Michelia skinnneriana	Magnoliaceae	Gutianshan	HQ427275	n/a	n/a
Miconia affinis	Melastomataceae	BCI	GQ981798	GQ982042	GQ982284
Miconia argentea	Melastomataceae	BCI	GQ981799	GQ982043	GQ982285
Miconia dorsiloba	Melastomataceae	BCI	GQ981800	BCI-M01	GQ982286
Miconia elata	Melastomataceae	BCI	GQ981801	n/a	GQ982287
Miconia hondurensis	Melastomataceae	BCI	GQ981802	n/a	GQ982288
Miconia impetiolaris	Melastomataceae	BCI	HM446827	GQ982044	HM446959
Miconia laevigata	Melastomataceae	Luquillo	HM446828	n/a	HM446960
Miconia mirabilis	Melastomataceae	Luquillo	HM446829	n/a	HM446961
Miconia nervosa	Melastomataceae	BCI	GQ981804	n/a	GQ982290
Miconia prasina	Melastomataceae	Luquillo	HM446830	n/a	HM446962
Miconia racemosa	Melastomataceae	Luquillo	HM446831	n/a	HM446963
Miconia tetrandra	Melastomataceae	Luquillo	HM446832	n/a	HM446964
Microcos blattaefolia	Malvaceae	Bukit-Timah	KJ594807	KJ709007	BTSIN445-13
Microcos paniculata	Malvaceae	Dinghushan	HQ415136	HQ415312	HQ415486
Microdesmis caseariifolia	Pandaceae	Dinghushan	HQ415208	HQ415373	HQ415555

Micropholis garciniifolia	Sapotaceae	Luquillo	HM446833	HM446713	HM446965
Micropholis guyanensis	Sapotaceae	Luquillo	HM446834	HM446714	HM446966
Mischocarpus pentapetalus	Sapindaceae	Dinghushan	HQ415054	HQ415242	HQ415406
Morella cerifera	Myricaceae	BCI	AF119179	EF590411	EF590715
Morinda seibertii	Rubiaceae	BCI	GQ981805	n/a	GQ982291
Morus alba	Moraceae	SERC	JN407320	n/a	GQ435324
Morus rubra	Moraceae	SERC	U06812	n/a	n/a
Mosannona garwoodii	Annonaceae	BCI	GQ981806	GQ982045	GQ982292
Mouriri myrtilloides	Melastomataceae	BCI	GQ981807	n/a	GQ982293
Myrcia deflexa	Myrtaceae	Luquillo	HM446835	HM446715	HM446967
Myrcia fallax	Myrtaceae	Luquillo	HM446836	HM446716	HM446968
Myrcia gatunensis	Myrtaceae	BCI	GQ981808	GQ982046	GQ982294
Myrcia leptoclada	Myrtaceae	Luquillo	HM446837	HM446717	HM446969
Myrcia splendens	Myrtaceae	Luquillo	HM446838	HM446718	HM446970
Myrica esculenta	Myricaceae	Bukit-Timah	KJ594810	n/a	n/a
Myrica rubra	Myricaceae	Fushan	KJ688436	HQ427396	HQ427096
Myristica cinnamomea	Myristicaceae	Bukit-Timah	KJ594811	KJ709009	BTSIN286-13
Myrospermum frutescens	Fabaceae	BCI	GQ981809	GQ982047	GQ982295
Myrsine coriacea	Myrsinaceae	Luquillo	HM446839	n/a	n/a
Nauclea officinalis	Rubiaceae	Dinghushan	HQ415201	n/a	n/a
Nectandra antillana	Lauraceae	Luquillo	GQ981812	n/a	HM446971
Nectandra cissiflora	Lauraceae	BCI	GQ981810	GQ982048	GQ982296
Nectandra fuzzy	Lauraceae	BCI	GQ982049	GQ981811	GQ982297
Nectandra lineata	Lauraceae	BCI	GQ982050	GQ981812	GQ982298
Nectandra purpurea	Lauraceae	BCI	BCI-R02	EU153858	EU153974
Nectandra umbrosa	Lauraceae	BCI	GQ981813	GQ982051	GQ982299
Neea amplifolia	Nyctaginaceae	BCI	GQ981814	GQ982052	GQ982300
Neolitsea aciculata var. variabillima	Lauraceae	Fushan	KJ688441	KJ687736	KJ687063
Neolitsea aurata	Lauraceae	Dinghushan	HQ415213	HQ415378	HQ415560
Neolitsea cambodiana	Lauraceae	Dinghushan	HQ415211	HQ415376	HQ415558
Neolitsea chui	Lauraceae	Dinghushan	HQ415210	HQ415375	HQ415557
Neolitsea konishii	Lauraceae	Fushan	KJ688446	KJ687737	KJ687072
Neolitsea umbrosa	Lauraceae	Dinghushan	HQ415212	HQ415377	HQ415559
Neoscortechinia forbesii	Euphorbiaceae	Bukit-Timah	KJ594815	KJ709013	BT-P12

Neoscortechinia kingii	Euphorbiaceae	Bukit-Timah	AB267912	AB268016	BTSIN649-13
Nephelium chryseum	Sapindaceae	Dinghushan	HQ415181	HQ415352	HQ415530
Nephelium costatum	Sapindaceae	Bukit-Timah	KJ594817	KJ709014	BTSIN465-13
Nephelium lappaceum	Sapindaceae	Bukit-Timah	KJ594819	KJ709016	BTSIN471-13
Nothaphoebe umbelliflora	Lauraceae	Bukit-Timah	KJ594822	KJ709020	BTSIN047-13
Nyssa sinensis	Cornaceae	Gutianshan	HQ427178	HQ427326	HQ427019
Nyssa sylvatica	Cornaceae	SERC	AF119178	GU266599	JQ280789
Ochanostachys amentacea	Olacaceae	Bukit-Timah	KJ594824	KJ709022	BT-P13
Ochroma pyramidale	Bombacaceae	Luquillo	HM446840	HM446719	GQ982301
Ocotea cernua	Lauraceae	BCI	GQ981816	JQ588119	GQ982302
Ocotea floribunda	Lauraceae	Luquillo	HM446841	HM446720	HM446972
Ocotea leucoxylon	Lauraceae	Luquillo	HM446842	HM446721	HM446973
Ocotea moschata	Lauraceae	Luquillo	HM446843	HM446722	HM446974
Ocotea oblonga	Lauraceae	BCI	GQ981817	GQ982053	GQ982303
Ocotea puberula	Lauraceae	BCI	GQ981818	GQ982054	GQ982304
Ocotea sintenisii	Lauraceae	Luquillo	HM446844	HM446723	HM446975
Ocotea spathulata	Lauraceae	Luquillo	HM446845	HM446724	HM446976
Ocotea whitei	Lauraceae	BCI	GQ981818	n/a	GQ982305
Oenocarpus mapora	Arecaceae	BCI	GQ981819	n/a	GQ982306
Oncosperma horridum	Arecaceae	Bukit-Timah	KJ594825	KJ709024	BTSIN486-13
Oreocnide pedunculata	Urticaceae	Fushan	KJ688452	n/a	KJ687073
Ormosia amazonica	Fabaceae	BCI	GQ981820	n/a	GQ982307
Ormosia coccinea	Fabaceae	BCI	GQ981821	GQ982055	GQ982308
Ormosia fordiana	Fabaceae:Papil.	Dinghushan	HQ415096	HQ415278	HQ415447
Ormosia formosana	Fabaceae	Lienhuachih	KJ688458	KJ687743	KJ687078
Ormosia glaberrima	Fabaceae:Papil.	Dinghushan	HQ415097	HQ415279	HQ415448
Ormosia krugii	Fabaceae:Papil.	Luquillo	HM446846	HM446725	HM446977
Ormosia macrocalyx	Fabaceae	BCI	GQ981822	GQ982056	GQ982309
Ormosia semicastrata	Fabaceae:Papil.	Dinghushan	HQ415098	HQ415280	HQ415449
Ormosia semicastrata f pallida	Fabaceae:Papil.	Dinghushan	HQ415095	HQ415277	HQ415446
Orophea creaghii	Annonaceae	Bukit-Timah	KJ594826	KJ709025	BTSIN443-13

Osmanthus cooperi	Oleaceae	Gutianshan	HQ427188	HQ427336	HQ427030
Osmanthus matsumuranus	Oleaceae	Fushan	KJ688459	KJ687744	KJ687079
Osmelia philippina	Salicaceae	Bukit-Timah	KJ594827	KJ709026	BTSIN298-13
Ostrya virginiana	Betulaceae	Wabikon Lake	KJ593568	KJ593034	FJ011865
Ouratea lucens	Ochnaceae	BCI	GQ981823	n/a	n/a
Oxandra laurifolia	Annonaceae	Luquillo	HM446847	HM446726	HM446978
Pachira quinata	Malvaceae	BCI	GQ981824	GQ982057	GQ982310
Pachira sessilis	Malvaceae	BCI	BCI-R03	n/a	n/a
Padus avium	Rosaceae	Changbaishan	CANGB027-14	n/a	n/a
Palaquium impressinervium	Sapotaceae	Bukit-Timah	KJ594829	n/a	n/a
Palaquium microphyllum	Sapotaceae	Bukit-Timah	n/a	KJ709027	n/a
Palaquium obovatum	Sapotaceae	Bukit-Timah	KJ594830	KJ709028	BTSIN351-13
Palaquium oxleyanum	Sapotaceae	Bukit-Timah	KJ594831	n/a	n/a
Palicourea guianensis	Rubiaceae	BCI	GQ981825	GQ982058	GQ982311
Palicourea riparia	Rubiaceae	Luquillo	HM446848	HM446727	HM446979
Pandanus sp.	Pandaceae	Bukit-Timah	KJ594832	KJ709029	n/a
Parartocarpus bracteatus	Moraceae	Bukit-Timah	KJ594833	n/a	BTSIN094-13
Parinari argentasericea	Chrysobalanaceae	Bukit-Timah	n/a	KJ709030	n/a
Parinari oblongifolia	Chrysobalanaceae	Bukit-Timah	JQ898743	KJ709031	BTSIN672-13
Parishia maingayi	Anacardiaceae	Bukit-Timah	KJ594834	KJ709032	BT-P14
Parishia paucijuga	Anacardiaceae	Bukit-Timah	KJ594835	KJ709033	BTSIN105-13
Parkia speciosa	Fabaceae	Bukit-Timah	KJ594837	KJ709035	BTSIN475-13
Pasania hancei var. ternaticupula	Fagaceae	Fushan	KJ688466	KJ687751	KJ687086
Pasania harlandii	Fagaceae	Fushan	KJ688474	KJ687756	KJ687093
Pasania konishii	Fagaceae	Lienhuachih	KJ688484	KJ687760	KJ687100
Pasania nantoensis	Fagaceae	Lienhuachih	KJ688485	KJ687761	KJ687101
Pasania synbalanos	Fagaceae	Lienhuachih	KJ688492	KJ687765	KJ687105
Paulownia tomentosa	Paulowniaceae	SCBI	L36447	AF051997	JF321288
Payena lucida	Sapotaceae	Bukit-Timah	KJ594838	KJ709037	BTSIN372-13
Pellacalyx	Rhizophoraceae	Bukit-Timah	KJ594842	KJ709040	BTSIN303-13

saccardianus					
Pentace triptera	Malvaceae	Bukit-Timah	KJ594844	KJ709042	BTSIN210-13
Pentagonia macrophylla	Rubiaceae	BCI	GQ981826	GQ982059	GQ982312
Pentaphylax euryoides	Pentaphylacaceae	Dinghushan	HQ415203	HQ415369	HQ415550
Perebea xanthochyma	Moraceae	BCI	GQ981827	GQ982060	GQ982313
Perrottetia arisanensis	Dipentodontaceae	Fushan	KJ688496	KJ687769	KJ687109
Pertusadina hainanensis	Rubiaceae	Gutianshan	HQ427197	HQ427346	HQ427040
Phellodendron amurense	Rutaceae	Changbaishan	CANGB028-14	CANGB028-15	CANGB028-16
Philadelphus schrenkii	Hydrangeaceae	Changbaishan	CANGB029-14	n/a	n/a
Photinia beauverdiana	Rosaceae	Gutianshan	HQ427204	HQ427353	HQ427047
Photinia glabra	Rosaceae	Gutianshan	HQ427205	HQ427354	n/a
Photinia parvifolia	Rosaceae	Gutianshan	HQ427206	HQ427355	n/a
Photinia prunifolia	Rosaceae	Dinghushan	HQ415183	n/a	HQ415532
Photinia serrulata	Rosaceae	Gutianshan	HQ427207	n/a	n/a
Phyllanthus glaucus	Euphorbiaceae	Gutianshan	HQ427130	n/a	n/a
Picea abies	Pinaceae	Wytham	NC_021456	NC_021456	NC_021456
Picea glauca	Pinaceae	Wabikon Lake	KJ593601	AF133926	JX508482
Picea jezoensis	Pinaceae	Changbaishan	CANGB030-14	CANGB030-15	CANGB030-16
Picea koraiensis	Pinaceae	Changbaishan	CANGB031-14	CANGB031-15	CANGB031-16
Picea mariana	Pinaceae	Wabikon Lake	KJ593605	EU364794	JX50849
Picramnia latifolia	Picramniaceae	BCI	GQ981828	n/a	GQ982314
Picrasma quassioides	Simaroubaceae	Gutianshan	HQ427179	HQ427327	HQ427020
Pieris formosa	Ericaceae	Gutianshan	HQ427148	HQ427301	HQ426993
Pieris japonica	Ericaceae	Gutianshan	HQ427151	HQ427303	HQ426995
Pimelodendron griffithianum	Euphorbiaceae	Bukit-Timah	KJ594847	n/a	n/a
Pinanga sp	Arecaceae	Bukit-Timah	KJ594849	KJ709045	n/a
Pinus koraiensis	Pinaceae	Changbaishan	CANGB032-14	CANGB032-15	CANGB032-16
Pinus massoniana	Pinaceae	Dinghushan	HQ427243	HQ427386	HQ427086

Pinus morrisonicola	Pinaceae	Lienhuachih	KJ688497	n/a	n/a
Pinus strobus	Pinaceae	Wabikon Lake	KJ593613	AY497255	EU750631
Pinus sylvestris var. sylvestriformis	Pinaceae	Changbaishan	CANGB033-14	n/a	n/a
Pinus taeda	Pinaceae	SERC	AF119177	AB080928	KJ661381
Pinus virginiana	Pinaceae	SERC	AB063379	AB080923	JQ512351
Piper aduncum	Piperaceae	Luquillo	HM446849	HM446728	HM446980
Piper aequale	Piperaceae	BCI	GQ981829	JQ588619	GQ982315
Piper arboreum	Piperaceae	BCI	n/a	GQ981830	GQ982316
Piper blattarum	Piperaceae	Luquillo	HM446850	HM446729	HM446981
Piper carrilloanum	Piperaceae	BCI	GQ981831	GQ982061	GQ982320
Piper colonense	Piperaceae	BCI	GQ981832	n/a	n/a
Piper cordulatum	Piperaceae	BCI	GQ981833	GQ982062	GQ982317
Piper glabrescens	Piperaceae	Luquillo	HM446851	HM446730	HM446982
Piper hispidum	Piperaceae	Luquillo	HM446852	DQ882219	HM446983
Piper multiplinervium	Piperaceae	BCI	JQ593229	JQ588651	n/a
Piper perlasense	Piperaceae	BCI	GQ981834	GQ982063	GQ982318
Piper reticulatum	Piperaceae	BCI	GQ981835	DQ882221	GQ982319
Piper umbellatum	Piperaceae	Luquillo	HM446853	GQ429067	HM446984
Pisonia subcordata	Nyctaginaceae	Luquillo	HM446854	HM446731	HM446985
Pithecellobium clypearia	Fabaceae:Mimos.	Dinghushan	HQ415100	HQ415281	HQ415451
Pittosporum glabratum	Pittosporaceae	Dinghushan	HQ415091	HQ415274	HQ415442
Pittosporum illicioides	Pittosporaceae	Gutianshan	HQ427157	HQ427307	HQ427000
Platycarya strobilacea	Juglandaceae	Gutianshan	HQ427158	HQ427308	HQ427001
Platymiscium pinnatum	Fabaceae	BCI	JQ626063	GQ982064	GQ982321
Pleodendron macranthum	Canellaceae	Luquillo	HM446855	HM446732	HM446986
Pluchea symphytifolia	Asteraceae	Luquillo	JQ590700	n/a	n/a
Podocarpus nakaii	Podocarpaceae	Lienhuachih	KJ688679	n/a	n/a
Polyalthia glauca	Annonaceae	Bukit-Timah	KJ594850	KJ709046	GQ248372
Polyalthia macropoda	Annonaceae	Bukit-Timah	KJ594852	KJ709047	n/a
Polyalthia rumphii	Annonaceae	Bukit-Timah	KJ594854	KJ709049	BTSIN103-13
Popowia pisocarpa	Annonaceae	Bukit-Timah	KJ594857	KJ709052	BTSIN453-13
Popowia tomentosa	Annonaceae	Bukit-Timah	KJ594858	KJ709053	BTSIN380-13

Populus balsamifera	Salicaceae	Wabikon Lake	KJ593626	KJ593064	n/a
Populus davidiana	Salicaceae	Changbaishan	CANGB034-14	n/a	n/a
Populus koreana	Salicaceae	Changbaishan	CANGB035-14	n/a	n/a
Populus tremuloides	Salicaceae	Wabikon Lake	KJ593631	KJ593068	n/a
Populus ussuriensis	Salicaceae	Changbaishan	CANGB036-14	n/a	n/a
Porterandia anisophylla	Rubiaceae	Bukit-Timah	KJ594859	KJ709054	BTSIN290-13
Posoqueria latifolia	Rubiaceae	BCI	GQ981837	GQ982066	GQ982323
Poulsenia armata	Moraceae	BCI	GQ981838	n/a	GQ982324
Pourouma bicolor	Urticaceae	BCI	GQ981839	GQ982067	GQ982325
Pourthiaea beauverdiana var. notabilis	Rosaceae	Fushan	KJ688498	KJ687770	KJ686637
Pouteria reticulata	Sapotaceae	BCI	GQ981841	JQ589190	GQ982327
Pouteria stipitata	Sapotaceae	BCI	GQ981842	GQ982069	GQ982328
Premna microphylla	Lamiaceae	Gutianshan	HQ427183	HQ427331	HQ427024
Prestoea montana	Arecaceae	Luquillo	HM446857	HM446733	HM446987
Prioria copaifera	Fabaceae	BCI	n/a	n/a	GQ982329
Prismatomeris glabra	Rubiaceae	Bukit-Timah	KJ594862	KJ709057	BTSIN275-13
Protium confusum	Burseraceae	BCI	GQ981843	GQ982070	GQ982330
Protium costaricense	Burseraceae	BCI	GQ981844	GQ982071	GQ982331
Protium panamense	Burseraceae	BCI	GQ981845	n/a	GQ982332
Protium tenuifolium	Burseraceae	BCI	GQ981846	n/a	GQ982333
Prunus avium	Rosaceae	Wytham	HQ235394	FJ899109	HQ188741
Prunus persica	Rosaceae	SCBI	NC_014697	NC_014697	NC_014697
Prunus polystachya	Rosaceae	Bukit-Timah	KJ594865	KJ709060	BTSIN251-13
Prunus schneideriana	Rosaceae	Gutianshan	HQ427209	HQ427356	HQ427052
Prunus serotina	Rosaceae	Wabikon Lake	KJ593640	KJ593077	DQ006222
Prunus serrulata	Rosaceae	Changbaishan	CANGB037-14	CANGB037-15	CANGB037-16
Prunus spinosa	Rosaceae	Wytham	n/a	HQ235276	FR865110
Prunus spinulosa	Rosaceae	Gutianshan	HQ427210	HQ427357	HQ427053
Prunus virginiana	Rosaceae	Wabikon Lake	KJ593643	KJ593079	GU562409

Pseudobombax septenatum	Malvaceae	BCI	GQ981847	GQ982072	GQ982334
Pseudolmedia spuria	Moraceae	Luquillo	HM446858	HM446734	HM446988
Pseudosamanea guachapele	Fabaceae	BCI	JQ591565	n/a	AF524983
Psidium friedrichsthalianum	Myrtaceae	BCI	GQ981848	GQ982073	GQ982335
Psychotria acuminata	Rubiaceae	BCI	GQ981849	GQ982074	GQ982336
Psychotria asiatica	Rubiaceae	Dinghushan	HQ415119	HQ415297	HQ415469
Psychotria berteroana	Rubiaceae	Luquillo	HM446859	HM446735	HM446989
Psychotria brachiata	Rubiaceae	Luquillo	HM446860	HM446736	HM446990
Psychotria chagrensis	Rubiaceae	BCI	GQ981850	GQ982075	GQ982337
Psychotria cyanococca	Rubiaceae	BCI	GQ981851	n/a	GQ982338
Psychotria deflexa	Rubiaceae	Luquillo	HM446861	HM446737	HM446991
Psychotria graciliflora	Rubiaceae	BCI	GQ981853	GQ982077	GQ982340
Psychotria grandis	Rubiaceae	Luquillo	HM446862	HM446738	HM446992
Psychotria hoffmannseggiana	Rubiaceae	BCI	GQ981853	n/a	n/a
Psychotria horizontalis	Rubiaceae	BCI	GQ981855	JQ589738	GQ982342
Psychotria limonensis	Rubiaceae	BCI	GQ981856	GQ982079	GQ982343
Psychotria marginata	Rubiaceae	BCI	GQ981857	GQ982080	GQ982344
Psychotria psychotriifolia	Rubiaceae	BCI	GQ981858	n/a	GQ982345
Psychotria racemosa	Rubiaceae	BCI	GQ981859	JQ589019	GQ982346
Psychotria rubra	Rubiaceae	Lienhuachih	KJ688513	n/a	KJ687121
Psychotria tenuifolia	Rubiaceae	BCI	GQ981860	GQ982081	GQ982347
Psydrax dicoccos	Rubiaceae	Dinghushan	HQ415205	HQ415371	HQ415571
Pternandra coerulescens	Melastomataceae	Bukit-Timah	n/a	KJ709061	BTSIN167-13
Pterocarpus belizensis	Fabaceae	BCI	HM446863	HM446739	HM446993

Pterocarpus officinalis	Fabaceae:Papil.	Luquillo	HM446863	HM446739	HM446993
Pterocarpus rohrii	Fabaceae	BCI	GQ981862	GQ982083	GQ982349
Pterospermum heterophyllum	Malvaceae	Dinghushan	HQ415057	HQ415245	HQ415409
Pygeum topengii	Rosaceae	Dinghushan	HQ415196	DQ851230	HQ415544
Pyrenaria shinkoensis	Theaceae	Fushan	KJ688514	KJ687781	KJ687122
Quararibea asterolepis	Malvaceae	BCI	n/a	n/a	GQ982350
Quararibea turbinata	Malvaceae	Luquillo	HM446864	HM446740	HM446994
Quassia amara	Simaroubaceae	BCI	GQ981863	n/a	GQ982351
Quercus alba	Fagaceae	SCBI	LM652853	LM652870	LM652974
Quercus argentata	Fagaceae	Bukit-Timah	KJ594869	KJ709063	BTSIN341-13
Quercus marilandica	Fagaceae	SCBI	KJ773806	KJ773054	SCBI-P01
Quercus michauxii	Fagaceae	SCBI	KJ773807	KJ773064	SCBI-P02
Quercus mongolica	Fagaceae	Changbaishan	CANGB038-14	CANGB038-15	CANGB038-16
Quercus muehlenbergii	Fagaceae	SCBI	KF683161	KR062090	SCBI-P03
Quercus phillyraeoides	Fagaceae	Gutianshan	HQ427176	HQ427324	HQ427012
Quercus robur	Fagaceae	Wytham	KF683161	FJ185056	FJ395542
Quercus rubra	Fagaceae	Wabikon Lake	KJ593650	KJ593082	EU750515
Quercus serrata	Fagaceae	Gutianshan	HQ427171	HQ427319	HQ427012
Quercus velutina	Fagaceae	SCBI	KJ593653	KJ593084	SCBI-P06
Radermachera sinica	Bignoniaceae	Lienhuachih	KJ688522	KJ687788	KJ687129
Randia armata	Rubiaceae	BCI	GQ981864	GQ982084	GQ982352
Randia cochinchinensis	Rubiaceae	Fushan	KJ688526	KJ687792	KJ687133
Rauvolfia littoralis	Apocynaceae	BCI	GQ981865	GQ982085	n/a
Rauvolfia nitida	Apocynaceae	Luquillo	DQ660663	n/a	n/a
Reevesia formosana	Malvaceae	Lienhuachih	KJ688530	n/a	n/a
Reevesia pycnantha	Malvaceae	Gutianshan	HQ427249	HQ427392	HQ427092
Reevesia thyrsoidea	Malvaceae	Dinghushan	HQ415190	HQ415360	HQ415539
Rhamnus crenata	Rhamnaceae	Gutianshan	HQ427242	HQ427385	HQ427085
Rhamnus ussuriensis	Rhamnaceae	Changbaishan	CANGB039-14	n/a	n/a
Rhaphiolepis indica	Rosaceae	Dinghushan	HQ415182	HQ415353	HQ415531

Rhaphiolepis indica var. tashiroi	Rosaceae	Lienhuachih	KJ688531	KJ687796	KJ687137
Rheedia portoricensis	Malpighiaceae	Luquillo	AF518377	n/a	M446995
Rhodamnia cinerea	Myrtaceae	Bukit-Timah	KJ594870	KJ709064	BTSIN149-13
Rhododendron henryi	Ericaceae	Dinghushan	HQ415074	HQ415258	HQ415425
Rhododendron henryi var. dunnii	Ericaceae	Dinghushan	HQ415075	HQ415259	HQ415426
Rhododendron latoucheae	Ericaceae	Gutianshan	HQ427145	HQ427298	HQ426990
Rhododendron leptosanthum	Ericaceae	Fushan	KJ688538	KJ687798	KJ687140
Rhododendron mariae	Ericaceae	Dinghushan	HQ415073	n/a	n/a
Rhododendron mariesii	Ericaceae	Lienhuachih	KJ688539	HQ427300	KJ687146
Rhododendron ovatum	Ericaceae	Gutianshan	HQ427144	HQ427297	HQ426989
Rhododendron simsii	Ericaceae	Dinghushan	HQ427146	HQ427299	HQ415427
Rhodomyrtus tomentosa	Myrtaceae	Dinghushan	HQ415191	HQ415361	HQ415540
Rhus hypoleuca	Anacardiaceae	Gutianshan	n/a	HQ427342	n/a
Rhus succedanea	Anacardiaceae	Fushan	KJ688544	KJ687802	KJ687150
Ribes mandshuricum	Grossulariaceae	Changbaishan	CANGB040-14	CANGB040-15	CANGB040-16
Rinorea sylvatica	Violaceae	BCI	GQ981866	GQ982086	GQ982353
Robinia pseudoacacia	Fabaceae	SCBI	DQ006096	HM049518	DQ006187
Rondeletia portoricensis	Rubiaceae	Luquillo	HM446866	HM446741	HM446996
Rosa macrophylla	Rosaceae	Changbaishan	CANGB041-14	n/a	n/a
Rosenbergiodendron formosum	Rubiaceae	BCI	GQ981867	GQ982087	GQ982354
Roystonea borinquena	Arecaceae	Luquillo	HM446867	HM446742	HM446997
Rubus allegheniensis	Rosaceae	SCBI	EU676983	EU749375	EU750524
Rubus chingii	Rosaceae	Gutianshan	HQ427211	HQ427358	HQ427054
Rubus pensilvanicus	Rosaceae	SCBI	EU676983	n/a	n/a
Rubus	Rosaceae	SCBI	JN965825	SCBI-M01	SCBI-P04

phoenicolasius					
Ryania speciosa	Salicaceae	BCI	HM446868	n/a	n/a
Sambucus canadensis	Adoxaceae	SCBI	HQ590258	HQ593429	HQ596833
Sambucus nigra	Adoxaceae	Wytham	KJ773864	FN668836	FN675824
Sambucus racemosa	Adoxaceae	Wabikon Lake	KJ593677	KJ593099	n/a
Sambucus williamsii	Adoxaceae	Changbaishan	CANGB042-14	n/a	n/a
Samyda dodecandra	Salicaceae	Luquillo	HM446868	HM446743	HM446998
Sandoricum beccarianum	Meliaceae	Bukit-Timah	KJ594872	KJ709065	n/a
Santiria griffithii	Burseraceae	Bukit-Timah	KJ594874	KJ709067	BTSIN594-13
Santiria rubiginosa	Burseraceae	Bukit-Timah	BTSIN655-13	n/a	BTSIN655-13
Santiria tomentosa	Burseraceae	Bukit-Timah	KJ594877	KJ709070	BTSIN622-13
Sapindus mukorossi	Sapindaceae	Lienhuachih	KJ688552	KJ687809	KJ687158
Sapium broadleaf	Euphorbiaceae	BCI	GQ982088	GQ981868	GQ982355
Sapium glandulosum	Euphorbiaceae	BCI	GQ981869	GQ982089	GQ982356
Sapium laurocerasus	Euphorbiaceae	Luquillo	HM446869	n/a	HM446999
Saprosma glomerulatum	Rubiaceae	Bukit-Timah	KJ594878	KJ709071	BTSIN273-13
Sarcosperma laurinum	Sapotaceae	Dinghushan	HQ415158	HQ415331	HQ415507
Sarcotheca griffithii	Oxalidaceae	Bukit-Timah	BT-R14	KJ709072	BTSIN603-13
Sassafras albidum	Lauraceae	SCBI	GU271217	EU153879	EF491223
Sassafras tzumu	Lauraceae	Gutianshan	HQ427277	HQ427419	HQ427120
Saurauia tristyla	Actinidiaceae	Dinghushan	n/a	EU310435	n/a
Scaphium macropodum	Malvaceae	Bukit-Timah	KJ594879	KJ709073	BTSIN339-13
Schefflera heptaphylla	Araliaceae	Dinghushan	HQ415082	KP093353	HQ415433
Schefflera morototoni	Araliaceae	Luquillo	HM446870	HM446744	HM447000
Schefflera octophylla	Araliaceae	Fushan	KJ688559	KJ687814	KJ687161
Schima superba	Theaceae	Lienhuachih	KJ688568	KJ687823	KJ687170
Schizolobium parahyba	Fabaceae	BCI	GQ981870	GQ982090	GQ982357
Schoepfia chinensis	Olacaceae	Dinghushan	HQ415145	HQ415320	HQ415495
Schoepfia jasminodora	Olacaceae	Dinghushan	HQ415146	HQ415321	HQ415496

Scleropyrum wallichianum	Santalaceae	Bukit-Timah	KJ594880	KJ709074	BTSIN272-13
Scorodocarpus borneensis	Olacaceae	Bukit-Timah	KJ594882	KJ709075	BTSIN266-13
Senna dariensis	Fabaceae	BCI	GQ981871	GQ982091	GQ982358
Shorea assamica	Dipterocarpaceae	Bukit-Timah	n/a	KJ709077	BTSIN581-13
Shorea curtisii	Dipterocarpaceae	Bukit-Timah	BTSIN109-13	n/a	BTSIN109-13
Shorea gratissima	Dipterocarpaceae	Bukit-Timah	KJ594886	n/a	BTSIN358-13
Shorea ochrophloia	Dipterocarpaceae	Bukit-Timah	BT-R15	KJ709081	BTSIN668-13
Shorea ovalis	Dipterocarpaceae	Bukit-Timah	n/a	KJ709082	BTSIN628-13
Shorea pauciflora	Dipterocarpaceae	Bukit-Timah	n/a	KJ709083	BTSIN580-13
Simarouba amara	Simaroubaceae	BCI	HM446871	JQ589560	HM447001
Sindora coriacea	Fabaceae	Bukit-Timah	KJ594887	KJ709084	BTSIN179-13
Siparuna guianensis	Siparunaceae	BCI	GQ981872	GQ982092	GQ982360
Siparuna pauciflora	Siparunaceae	BCI	GQ981873	GQ982093	GQ982361
Sloanea berteroana	Elaeocarpaceae	Luquillo	HM446872	HM446745	HM447002
Sloanea sinensis	Elaeocarpaceae	Dinghushan	HQ427152	n/a	n/a
Sloanea terniflora	Elaeocarpaceae	BCI	GQ981874	GQ982094	GQ982362
Socratea exorrhiza	Arecaceae	BCI	GQ981875	GQ982095	GQ982363
Solanum asperum	Solanaceae	BCI	GQ981876	GQ982096	GQ982364
Solanum circinatum	Solanaceae	BCI	GQ981877	GQ982097	GQ982365
Solanum hayesii	Solanaceae	BCI	GQ981878	GQ982098	GQ982366
Solanum lepidotum	Solanaceae	BCI	GQ981879	GQ982099	GQ982367
Solanum rugosum	Solanaceae	Luquillo	JQ594175	JQ589269	n/a
Sorbaria sorbifolia	Rosaceae	Changbaishan	CANGB043-14	CANGB043-15	CANGB043-16
Sorbus alnifolia	Rosaceae	Changbaishan	CANGB044-14	n/a	n/a
Sorbus folgneri	Rosaceae	Gutianshan	HQ427212	HQ427359	HQ427055
Sorbus pohuashanensis	Rosaceae	Changbaishan	CANGB045-14	n/a	n/a
Sorocea affinis	Moraceae	BCI	GQ981880	GQ982100	GQ982368
Spachea membranacea	Malpighiaceae	BCI	GQ981881	GQ982101	GQ982369
Spathodea campanulata	Bignoniaceae	Luquillo	HM446873	HM446746	HM447003
Spiraea chamaedryfolia	Rosaceae	Changbaishan	CANGB046-14	n/a	n/a
Spondias mombin	Anacardiaceae	BCI	GQ981882	AY594480	GQ982370
Spondias radlkoferi	Anacardiaceae	BCI	GQ981883	n/a	GQ982371
Stemmadenia grandiflora	Apocynaceae	BCI	GQ981884	GQ982102	GQ982372

Stemonurus malaccensis	Stemonuraceae	Bukit-Timah	BTSIN154-13	n/a	BTSIN154-13
Sterculia apetala	Malvaceae	BCI	GQ981885	GQ982103	GQ982373
Sterculia coccinea	Malvaceae	Bukit-Timah	KJ594892	KJ709089	BTSIN477-13
Sterculia lanceolata	Malvaceae	Dinghushan	HQ415135	HQ415311	HQ415485
Sterculia parviflora	Malvaceae	Bukit-Timah	KJ594896	KJ709092	BTSIN289-13
Sterculia rubiginosa	Malvaceae	Bukit-Timah	KJ594897	KJ709093	n/a
Stereospermum fimbriatum	Bignoniaceae	Bukit-Timah	BT-R16	KJ709094	BTSIN584-13
Streblus elongatus	Moraceae	Bukit-Timah	BTSIN085-13	n/a	BTSIN085-13
Strombosia ceylanica	Olacaceae	Bukit-Timah	KJ594898	KJ709095	n/a
Stylogyne turbacensis	Primulaceae	BCI	GQ981886	GQ982104	GQ982374
Styrax dasyanthus	Styracaceae	Gutianshan	HQ427123	HQ427280	HQ426970
Styrax formosanus	Styracaceae	Fushan	KJ688573	KJ687826	KJ687175
Styrax odoratissimus	Styracaceae	Gutianshan	HQ427125	HQ427282	HQ426972
Styrax suberifolius	Styracaceae	Dinghushan	HQ427124	HQ427281	HQ415402
Swartzia simplex	Fabaceae	BCI	GQ981887	GQ982105	n/a
Swartzia simplex var. grandiflora	Fabaceae	BCI	GQ981887	GQ982105	n/a
Swartzia simplex var. ochnacea	Fabaceae	BCI	GQ981887	GQ982105	n/a
Swietenia macrophylla	Meliaceae	Luquillo	HM446874	HM446747	HM447004
Swintonia schwenckei	Anacardiaceae	Bukit-Timah	KJ594901	n/a	BTSIN436-13
Symphonia globulifera	Clusiaceae	BCI	GQ981889	GQ429061	GQ982376
Symplocos adenopus	Symplocaceae	Dinghushan	HQ415169	HQ415340	HQ415518
Symplocos adinandrifolia var. theifolia	Symplocaceae	Lienhuachih	KJ688585	KJ687835	KJ687187
Symplocos anomala	Symplocaceae	Gutianshan	HQ427233	n/a	n/a
Symplocos caudata	Symplocaceae	Fushan	KJ688586	KJ687836	KJ687188
Symplocos glauca	Symplocaceae	Fushan	KJ688592	KJ687842	KJ687194
Symplocos heishanensis	Symplocaceae	Fushan	KJ688599	FS-M01	KJ687201
Symplocos lancifolia	Symplocaceae	Dinghushan	HQ415167	HQ415339	HQ415516
Symplocos	Symplocaceae	Luquillo	HM446875	AY630657	HM447005

martinicensis					
Symplocos morrisonicola	Symplocaceae	Lienhuachih	KJ688604	KJ687849	KJ687204
Symplocos paniculata	Symplocaceae	Gutianshan	HQ427234	HQ427378	HQ427077
Symplocos setchuensis	Symplocaceae	Fushan	KJ688608	KJ687853	KJ687208
Symplocos sonoharae	Symplocaceae	Fushan	KJ688613	n/a	KJ687213
Symplocos stellaris	Symplocaceae	Gutianshan	HQ427236	HQ427379	HQ427078
Symplocos sumuntia	Symplocaceae	Gutianshan	HQ427232	HQ427377	HQ427075
Symplocos theophrastifolia	Symplocaceae	Nanjenshan	KJ688847	KJ687959	KJ687437
Symplocos wikstroemiifolia	Symplocaceae	Fushan	KJ688623	KJ687866	KJ687222
Syringa reticulata subsp amurensis	Oleaceae	Changbaishan	CANGB047-14	n/a	n/a
Syringa wolfii	Oleaceae	Changbaishan	CANGB048-14	n/a	n/a
Syzygium acuminatissimum	Myrtaceae	Dinghushan	HQ415209	HQ415374	HQ415556
Syzygium brittle	Myrtaceae	Bukit-Timah	n/a	KJ709097	n/a
Syzygium buxifolirm	Myrtaceae	Gutianshan	HQ427244	HQ427387	HQ427087
Syzygium championii	Myrtaceae	Dinghushan	HQ415142	HQ415318	HQ415492
Syzygium duthieanum	Myrtaceae	Bukit-Timah	KJ594903	KJ709098	BT-P15
Syzygium filiforme	Myrtaceae	Bukit-Timah	BT-R17	KJ709099	BTSIN620-13
Syzygium filiforme	Myrtaceae	Bukit-Timah	n/a	KJ709099	BTSIN620-13
Syzygium formosanum	Myrtaceae	Fushan	KJ688630	KJ687869	KJ687227
Syzygium grande	Myrtaceae	Bukit-Timah	n/a	KJ709101	n/a
Syzygium hancei	Myrtaceae	Dinghushan	HQ415140	HQ415316	HQ415490
Syzygium jambos	Myrtaceae	Luquillo	HM446876	HM446748	HM447006
Syzygium levinei	Myrtaceae	Dinghushan	HQ415137	HQ415313	HQ415487
Syzygium linocieroideum	Myrtaceae	Bukit-Timah	BTSIN633-13	n/a	BTSIN633-13
Syzygium nigricans	Myrtaceae	Bukit-Timah	KJ594909	KJ709107	BT-P16
Syzygium oblongifolium	Myrtaceae	Bukit-Timah	n/a	n/a	n/a
Syzygium palembanicum	Myrtaceae	Bukit-Timah	n/a	KJ709108	n/a
Syzygium pauper	Myrtaceae	Bukit-Timah	n/a	KJ709109	n/a

Syzygium pendens	Myrtaceae	Bukit-Timah	n/a	KJ709110	n/a
Syzygium pseudoformosum	Myrtaceae	Bukit-Timah	KJ594910	KJ709111	BTSIN422-13
Syzygium rehderianum	Myrtaceae	Dinghushan	HQ415139	HQ415315	HQ415489
Syzygium ridleyi	Myrtaceae	Bukit-Timah	KJ594912	KJ709116	n/a
Syzygium singaporense	Myrtaceae	Bukit-Timah	KJ594913	KJ709117	n/a
Syzygium subdecussatum	Myrtaceae	Bukit-Timah	n/a	KJ709118	BTSIN123-13
Syzygium zeylanicum	Myrtaceae	Bukit-Timah	KJ594914	n/a	n/a
Tabebuia guayacan	Bignoniaceae	BCI	GQ981890	GQ982107	GQ982377
Tabebuia heterophylla	Bignoniaceae	Luquillo	HM446877	HQ384521	HM447007
Tabebuia rosea	Bignoniaceae	BCI	GQ981891	GQ982108	GQ982378
Tabernaemontana arborea	Apocynaceae	BCI	GQ981892	GQ982109	GQ982379
Tachigali versicolor	Fabaceae	BCI	GQ981893	n/a	GQ982380
Talisia croatii	Sapindaceae	BCI	GQ981894	n/a	GQ982381
Tarenna costata	Rubiaceae	Bukit-Timah	KJ594915	KJ709119	BTSIN429-13
Tarenna mollissima	Rubiaceae	Dinghushan	HQ427200	HQ427349	HQ415548
Taxus baccata	Taxaceae	Wytham	NC_020321	NC_020321	NC_020321
Teijsmanniodendron coriaceum	Lamiaceae	Bukit-Timah	KJ594916	KJ709120	BTSIN120-13
Terminalia amazonia	Combretaceae	BCI	GQ981895	n/a	GQ982382
Terminalia citrina	Combretaceae	Bukit-Timah	n/a	n/a	n/a
Terminalia oblonga	Combretaceae	BCI	GQ981896	n/a	GQ982383
Tetradium glabrifolium	Rutaceae	Fushan	KJ688636	KJ687871	KJ687232
Tetragastris balsamifera	Burseraceae	Luquillo	HM446878	HM446749	HM447008
Tetragastris panamensis	Burseraceae	BCI	GQ428579	n/a	GQ982384
Tetrathylacium johansenii	Salicaceae	BCI	GQ981898	GQ982110	GQ982385
Theobroma cacao	Malvaceae	BCI	GQ981897	GQ982111	GQ982386
Thevetia ahouai	Apocynaceae	BCI	GQ981899	GQ982112	GQ982387
Thuja occidentalis	Cupressaceae	Wabikon Lake	KJ593720	KJ661406	KJ661394
Tilia americana	Malvaceae	Wabikon Lake	KJ593722	KJ593130	HQ596866

Tilia amurensis	Malvaceae	Changbaisha n	CANGB049-14	n/a	n/a
Tilia endochrysea	Malvaceae	Gutianshan	HQ427156	HQ427306	n/a
Tilia mandshurica	Malvaceae	Changbaisha n	CANGB050-14	n/a	n/a
Tocoyena pittieri	Rubiaceae	BCI	GQ981900	GQ982113	GQ982388
Toxicodendron succedaneum	Anacardiaceae	Dinghushan	HQ427194	HQ427343	n/a
Toxicodendron sylvestre	Anacardiaceae	Dinghushan	HQ415143	HQ415319	HQ415493
Trattinnickia aspera	Burseraceae	BCI	GQ981901	GQ982114	GQ982389
Trema cannabina	Rosaceae	Lienhuachih	KJ688640	KJ687875	KJ687238
Trema micrantha	Ulmaceae	Luquillo	KJ082620	KJ012809	GQ982390
Trema tomentosa	Ulmaceae	Dinghushan	HQ415174	HQ415345	HQ415523
Triadica cochinchinensis	Euphorbiaceae	Dinghushan	HQ415199	HQ415366	HQ415547
Tricalysia dubia	Rubiaceae	Fushan	KJ688647	KJ687881	KJ687244
Trichanthera gigantea	Acanthaceae	BCI	GQ981903	GQ982116	GQ982391
Trichilia pallida	Meliaceae	Luquillo	HM446879	HM446750	HM447009
Trichilia tuberculata	Meliaceae	BCI	GQ981905	n/a	GQ982393
Trichospermum galeottii	Malvaceae	BCI	JQ594273	n/a	n/a
Trigonostemon longifolius	Euphorbiaceae	Bukit-Timah	KJ594921	KJ709124	n/a
Triomma malaccensis	Burseraceae	Bukit-Timah	KJ594922	KJ709125	n/a
Triplaris cumingiana	Polygonaceae	BCI	GQ981906	GQ982118	GQ982394
Trophis caucana	Moraceae	BCI	GQ981907	GQ982119	GQ982395
Trophis racemosa	Moraceae	BCI	GQ981908	GQ982120	GQ982396
Tsuga canadensis	Pinaceae	Wabikon Lake	KJ593735	JQ512502	JQ512378
Turpinia occidentalis	Staphyleaceae	Luquillo	HM446880	HM446751	HM447010
Tutcheria microcarpa	Theaceae	Gutianshan	HQ427231	HQ427376	HQ427387
Ulmus americana	Ulmaceae	Wabikon Lake	KJ593739	KJ593141	n/a
Ulmus davidiana	Ulmaceae	Changbaisha n	CANGB051-14	n/a	n/a
Ulmus laciniata	Ulmaceae	Changbaisha	CANGB052-14	n/a	n/a

		n			
Ulmus rubra	Ulmaceae	SCBI	KC539680	KC539611	SCBI-P05
Unonopsis pittieri	Annonaceae	BCI	GQ981910	GQ982122	GQ982398
Urera baccifera	Urticaceae	Luquillo	HM446881	HM446752	HM447011
Urophyllum glabrum	Rubiaceae	Bukit-Timah	KJ594924	KJ709127	BTSIN206-13
Urophyllum hirsutum	Rubiaceae	Bukit-Timah	KJ594925	KJ709128	BT-P17
Vaccinium bracteatum	Ericaceae	Fushan	KJ688660	KP093519	KJ687256
Vaccinium randaiense	Ericaceae	Lienhuachih	KJ688664	n/a	KJ687261
Vachellia melanoceras	Fabaceae	BCI	GQ981912	GQ982124	GQ982400
Vatica odorata	Dipterocarpaceae	Bukit-Timah	AB925379	KJ709131	BTSIN624-13
Vatica ridleyana	Dipterocarpaceae	Bukit-Timah	KJ594928	KJ709132	BTSIN416-13
Verbesina gigantea	Asteraceae	BCI	JQ590731	JQ586939	n/a
Vernicia montana	Euphorbiaceae	Gutianshan	HQ427131	HQ427287	HQ426977
Viburnum acerifolium	Adoxaceae	SCBI	DQ006079	n/a	DQ006166
Viburnum acerifolium	Caprifoliaceae	SCBI	KJ593749	HQ591557	KJ795522
Viburnum burejaeticum	Caprifoliaceae	Changbaishan	CANGB053-14	n/a	n/a
Viburnum erosum	Caprifoliaceae	Gutianshan	HQ427216	HQ427362	HQ427059
Viburnum formosanum	Adoxaceae	Fushan	KJ688668	n/a	KJ687265
Viburnum luzonicum	Adoxaceae	Lienhuachih	KJ688669	KP094047	KJ687266
Viburnum odoratissimum	Adoxaceae	Dinghushan	HQ415114	HQ415292	HQ415464
Viburnum prunifolium	Adoxaceae	SCBI	DQ006080	KP089365	DQ006167
Viburnum sargentii	Adoxaceae	Changbaishan	CANGB054-14	n/a	n/a
Viburnum sempervirens	Adoxaceae	Gutianshan	HQ427217	HQ427363	HQ427060
Virola multiflora	Myristicaceae	BCI	GQ981913	GQ982125	GQ982401
Virola nobilis	Myristicaceae	BCI	GQ981914	GQ982126	GQ982402
Virola sebifera	Myristicaceae	BCI	GQ981915	n/a	n/a
Vismia baccifera	Hypericaceae	BCI	GQ981916	n/a	GQ982403
Vismia billbergiana	Hypericaceae	BCI	GQ981917	GQ982127	GQ982404
Vitex divaricata	Verbenaceae	Luquillo	U78716	n/a	HM447012
Vitex quinata	Verbenaceae	Dinghushan	HQ415126	HQ415304	HQ415476

Walsura chrysogyne	Meliaceae	Bukit-Timah	KJ594932	KJ709133	BTSIN450-13
Weigela japonica	Caprifoliaceae	Gutianshan	HQ427218	HQ427364	HQ427061
Wendlandia formosana	Rubiaceae	Fushan	KJ688671	KJ687895	KJ687269
Wendlandia uvariifolia	Rubiaceae	Lienhuachih	KJ688677	n/a	KJ687277
Wikstroemia indica	Thymelaeaceae	Dinghushan	HQ415147	HQ415322	HQ415497
Wikstroemia monnula	Thymelaeaceae	Gutianshan	HQ427215	n/a	n/a
Wikstroemia nutans	Thymelaeaceae	Dinghushan	HQ415148	n/a	HQ415498
Xanthophyllum amoenum	Polygalaceae	Bukit-Timah	BTSIN557-13	n/a	BTSIN557-13
Xanthophyllum ellipticum	Polygalaceae	Bukit-Timah	BTSIN141-13	n/a	BTSIN141-13
Xanthophyllum eurhynchum	Polygalaceae	Bukit-Timah	KJ594935	KJ709135	BTSIN310-13
Xanthophyllum griffithii	Polygalaceae	Bukit-Timah	KJ594936	n/a	BTSIN350-13
Xanthophyllum hainanense	Polygalaceae	Dinghushan	HQ415112	HQ415290	HQ415462
Xerospermum noronhianum	Sapindaceae	Bukit-Timah	KJ594938	KJ709136	BTSIN062-13
Xylopia caudata	Annonaceae	Bukit-Timah	KJ594940	KJ709137	BTSIN464-13
Xylopia macrantha	Annonaceae	BCI	n/a	n/a	GQ982406
Xylopia malayana	Annonaceae	Bukit-Timah	BTSIN190-13	n/a	BTSIN190-13
Xylosma oligandra	Salicaceae	BCI	GQ981919	GQ982129	GQ982407
Xylosma racemosa	Salicaceae	Gutianshan	HQ427273	n/a	n/a
Xylosma schwaneckeana	Salicaceae	Luquillo	HM446882	HM446753	HM447013
Zanthoxylum ailanthoides	Rutaceae	Lienhuachih	KJ688678	KJ687897	KJ687278
Zanthoxylum avicennae	Rutaceae	Dinghushan	HQ415109	HQ415288	HQ415459
Zanthoxylum ekmanii	Rutaceae	BCI	GQ981920	GQ982130	GQ982408
Zanthoxylum juniperinum	Rutaceae	BCI	GQ981921	BCI-M03	GQ982409
Zanthoxylum martinicense	Rutaceae	Luquillo	HM446883	HM446754	HM447014
Zanthoxylum myriacanthum	Rutaceae	Dinghushan	HQ415108	HQ415287	HQ415458
Zanthoxylum panamense	Rutaceae	BCI	n/a	GQ981922	GQ982410

Zanthoxylum setulosum	Rutaceae	BCI	GQ981923	GQ982131	GQ982411
Zuelania guidonia	Salicaceae	BCI	GQ981924	n/a	GQ982412
